# Scanning Tunneling Microscope-Based Break-Junction Technique—A Tutorial

**DOI:** 10.1021/acsphyschemau.6c00026

**Published:** 2026-05-04

**Authors:** Emma York, Latha Venkataraman

**Affiliations:** † Department of Chemistry, 5798 Columbia University, New York, New York 10027, United States; ‡ Institute of Science and Technology Austria, 3400 Klosterneuburg, Austria; § Department of Applied Physics and Applied Mathematics, Columbia University, New York, New York 10027, United States

**Keywords:** single-molecule electronics, STM break-junction, molecular junctions, molecular conductance, charge transport, statistical analysis, conductance histograms, instrumentation, experimental methods

## Abstract

Over the past two decades, molecular electronics has made significant progress toward discovering nanoscale analogues of conventional electronic components, largely enabled by the development of the scanning tunneling microscope-based break-junction (STM-BJ) technique. The STM-BJ technique enables precise and highly reproducible measurement of a molecule’s electronic transport properties, making it a powerful technique to explore physiochemical and electrochemical phenomena that are otherwise difficult to access. It has gained substantial popularity in the past 20 years, with experiments becoming increasingly diverse and sophisticated. Despite the wealth of literature, an accessible, practical guide to performing STM-BJ experiments and interpreting the data is largely absent. This tutorial includes a brief background into the development of STM-BJ measurements, followed by detailed explanations of instrumentation, data collection, statistical analysis, variations on standard experiments, and some troubleshooting methods. It is aimed at researchers looking to begin or improve STM-BJ studies in their laboratories, graduate students and postdoctoral researchers learning the technique, and readers seeking to critically evaluate the growing body of STM-BJ literature.

## Graphical abstract

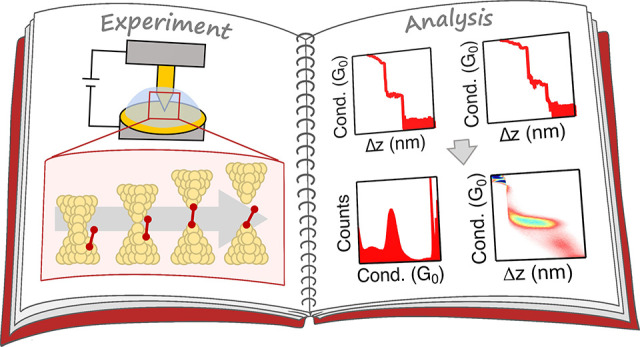

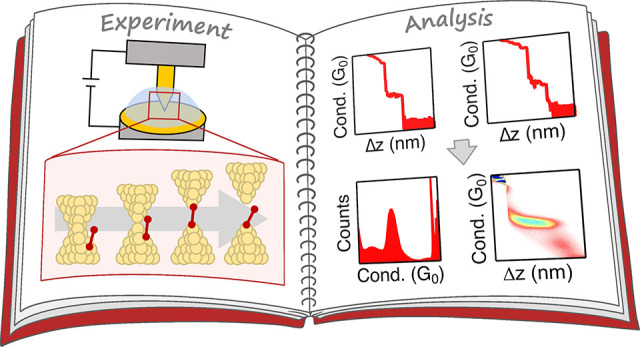

Over the past two decades, molecular electronics has made significant progress toward discovering nanoscale analogues of conventional electronic components—including resistive elements,^1–6^ diodes,^7–11^ switches,^12–19^ transistors,^20–25^ and more recently, highly conducting wires.^26–28^ These advances have been largely enabled by the development of the scanning tunneling microscope-based break-junction (STM-BJ) technique,^29–32^ in which a thin metallic contact between the STM tip and substrate is broken in the presence of target molecules, allowing a single molecule to bridge the resulting gap. The STM-BJ technique enables precise and highly reproducible measurement of the molecule’s electronic transport properties, making it a powerful technique to explore physiochemical and electrochemical phenomena that are otherwise difficult to access.

The STM-BJ technique was first developed by Agraït and co-workers in 1993 ^33^ to study metal point contacts and was then used by Xu and Tao in 2003 ^3^ to demonstrate conductance measurements in single-molecule devices. It has gained substantial popularity in the last 20 years, with experiments becoming increasingly diverse and sophisticated. Numerous reviews have detailed methodological advances as well as scientific insights in nanoscale physics, chemistry, and quantum transport.^34–39^ Despite the wealth of literature, an accessible, practical guide to performing STM-BJ experiments and interpreting the data is largely absent. This tutorial includes a brief background into the development of STM-BJ measurements, followed by detailed explanations of instrumentation, data collection, statistical analysis, variations on standard experiments, and some troubleshooting methods. It is aimed at researchers looking to begin or improve STM-BJ studies in their laboratories, graduate students and postdoctoral researchers learning the technique, and readers seeking to critically evaluate the growing body of STM-BJ literature.

## 1 Background

Some of the first breakthroughs and fundamental insights into charge transport in atom-scale metallic systems were achieved with the mechanically controlled break-junction (MCBJ) technique, including the first demonstration of quantized conductance phenomena in a metallic system.^29,40^ In this method, a metallic wire containing a narrow constriction is mounted on a flexible substrate and bent using a piezoelectric actuator. While the constriction thins to single-atom dimensions and eventually breaks, a voltage (V) is applied across the wire and current (I) is measured as a function of displacement. Reversing the bending restores contact, enabling conductance measurements over thousands of breaking and rejoining cycles. Experiments utilizing the MCBJ technique revealed that the conductance of Au, Ag, and Cu atomic contacts is quantized in units of *G*_0_ = 2*e* ^2^/*h*, where *e* is the charge of the electron and *h* is Planck’s constant. This is consistent with the electronic configuration of Group 11 metals, where the *d* orbitals are fully occupied and a singly occupied *s* orbital provides a single, fully transmitting channel for conduction. In contrast, other metal atomic contacts (e.g., Pt, Al, Pb, Nb) have several channels that are only partially open, reflecting the contributions from their partially filled *s*, *p*, and/or *d* orbitals.^41–43^

In 1993, Agraït and colleagues adapted the BJ method for use within an STM setup.^33^ In the STM-BJ technique, the STM tip is brought into and out of contact with the substrate under an applied bias, while the current is continuously measured as a function of the tip–sample displacement. This allows the generation of conductance–displacement traces analogous to those obtained in MCBJ experiments. Using this approach, Agraït et al. reported clear signatures of conductance quantization in Au and Pb point contacts.^33^ Measurements of the conductance of single-molecule junctions were first made using the MCBJ method in 1997 when Reed et al.^2^ recorded current–voltage characteristics across a gold nanogap coated with 1,4-benzenedithiol. This was soon followed by experiments measuring a range of molecules from H_2_ to complex organic systems.^31,44–49^ Molecular conductance was also measured in electromigrated break-junctions,^50–53^ a similar technique in which a current is used to break a micron-scale notched metal wire as opposed to mechanical bending.^20,54^

In 2003, Xu and Tao were the first to introduce molecules into STM-based break junctions, measuring the conductance of alkanedithiols and 4,4-bipyridine.^3^ After separating Au electrodes in a solution of target molecules, sub-*G*_0_ conductance steps were observed in conductance-displacement traces, corresponding to molecular bridges formed by S–Au and N–Au coordination. In 2006, Venkataraman et al. demonstrated that amine-terminated molecules yield highly reproducible conductance signatures,^55^ enabling numerous systematic studies mapping the relationships between molecular structure and charge transport.^56^ Following these foundational experiments, the STM-BJ technique has been widely used to probe structure–function relationships and to explore numerous chemical and physical phenomena at the single-molecule level including quantum interference,^57–60^ thermoelectric effects,^61–63^ electrochemical switching and gating,^64,65^ reactivity,^66–70^ and optoelectronic function,^71–73^ to name a few. STM-BJ offers numerous unique advantages in comparison to similar techniques. The experimental setup is straightforward and easily implemented using low-cost, commercial electronic parts. No complex tip fabrication is required and measurements can be performed under ambient conditions in air or solvent. This simplicity combined with the robust, stable junction environment allows high throughput; thousands of junctions can be measured over a period of hours to ensure statistically meaningful results. Since measurements are solution-compatible, diverse structures can be explored with minimal sample quantities (1 mg or less). The method is also highly customizable and can be coupled with other microscopy- and spectroscopy-based tools.

Results from conductance experiments can be compared with density functional theory (DFT) calculations to explain trends across molecular families and understand the impact of a molecule’s electronic structure on the measured conductance. This enables the identification of the nature of charge transport (e.g., electron or hole transport), and the impact of quantum interference effects on conductance enhancement or suppression. Conductance originates from electron transmission between the two electrodes; it is governed by the Landauer formalism and is determined through calculations of transmission probability. Transmission depends on the extent to which the molecular orbitals are coupled to the metal electrode (broadening or narrowing the resonances), and the energetic alignment between these orbitals and the electrode Fermi level. When the dominant transport orbitals (i.e., those energetically closest and coupled to the electrode) are energetically far from the Fermi energy, transport is through a coherent off-resonance mechanism; if a dominant orbital falls within the experimental bias window, conductance is through a resonant mechanism (Figure 1b).^74^

**Figure 1:**
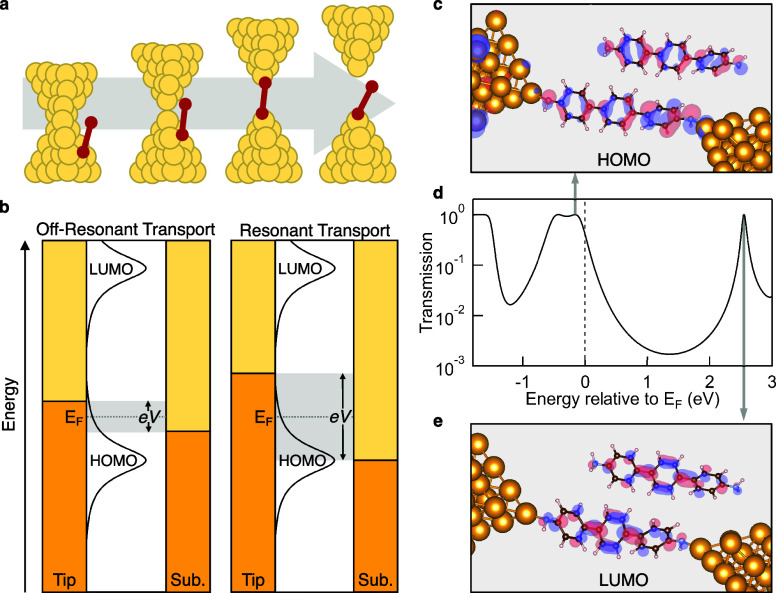
a) Illustration of the forming and breaking of a single-molecule junction: electrodes are brought into contact, retracted to establish a single-atom Au point contact, further separated to form a Au-molecule-Au junction, and finally separated completely. b) Schematic depicting on- and off-resonant transport regimes. In a low bias measurement, conducting orbitals are typically far from the experimental bias window (left panel). When the bias is increased and a conducting orbital can be accessed directly, resonant transport occurs (right panel). In this scheme, the bias window opens symmetrically about *E*_F_, as is expected in an air/nonpolar solvent environment (see Part [Sec sec6]). c–e) DFT-based transmission calculations for 4,4′′-diamino-*p*-terphenyl (**pTDA**). The transmission function shown in (d) is calculated for a single **pTDA** molecule bound to 2 × 22-atom model electrodes. The eigenstates at the resonances close to *E*_F_ closely match the orbital isosurface calculations of HOMO (c) and LUMO (e) for the isolated molecule.

As an example, we illustrate calculated transmission across a molecular junction of 4,4′′-diamino-*p*-terphenyl (**pTDA**) bound between two Au electrodes in Figure 1d. The transmission function is calculated using the nonequilibrium Green’s function (NEGF) method as implemented in the FHI-aims software package with the PBE functional.^75–79^ We follow a standard procedure in which we first optimize the geometry of the isolated molecule with a single Au atom attached at each nitrogen atom. We next append Au_21_ clusters onto the Au atom at each side to mimic the electrodes, and then compute the transmission using the AITRANSS package.^75–77^ Results from these calculations are shown in Figure 1c-e, highlighting the orbitals responsible for transmission, along with eigenstates at the resonances. We compare these eigenstates with isosurface calculations of the frontier orbitals in an isolated molecule (Figure 1c and 1e), where we see a strong similarity.

Since the current is proportional to the integral of the transmission curve within the applied bias window, conductance trends across multiple molecules can be evaluated by comparing the transmission at the Fermi energy. These calculations that use low-rung functionals (e.g., PBE) overestimate the conductance relative to experiments due to errors inherent to the DFT-based method^75,77^ and are therefore used to examine trends in experiments rather than for quantitative accuracy. Further details on transport calculations can be found in reviews elsewhere.^34,80^

## 2 Instrument Design

A unique and attractive benefit of the STM-BJ technique is that the instrument can be constructed from commercially available parts. While instrument details in individual laboratories may vary, the fundamental setup requires moving the STM tip relative to the substrate with sub-angstrom precision, applying a voltage across the molecular junction, and measuring the pA-μA scale junction current at kilohertz speeds to determine the conductance. The motion and simultaneous measurements of junction current and voltage are controlled through an automated experimental procedure, enabling the collection of thousands of conductance versus displacement traces for statistical analysis without data selection. The equipment required for such a setup is listed below, including the exact parts used in our lab.

(1) Tip and sample holders (custom).
(2) Piezoelectric actuator to control tip/substrate relative motion with sub-angstrom precision (Mad City Laboratories HSZ or Physik Instrumente P-840.10).
(3) Coarse-approach motor to bring the tip close to the substrate within the travel range of the piezo (Newport NSA12 or Newport Agilis AG-LS25).
(4) Current amplifier to convert pA-scale junction currents to measurable voltage signals (Keithley 428-PROG or Femto DLPCA-200 or NF Corporation CA5350).
(5) A clean, low-noise voltage source for the junction bias and piezo voltage (National Instruments Data Acquisition Card PXI-4461 or PXI-4468).
(6) Data acquisition system to collect data and allow communication between the instrument and computer (National Instruments Data Acquisition Card PXI-4461 or PXI-4468).
(7) Vibration isolation for the setup such as a bungee system, an active isolation table (HWL Scientific TS-150), or an air table (may require an additional layer of vibration isolation materials).
(8) Acoustic/electrical isolation chamber (Faraday cage) to prevent noise from interfering with the measurement (custom).

A photograph of an example STM-BJ setup is shown in Figure 2. A segment of Au wire with a diameter of 250 μm (Fisher Scientific 11337058, 99.998% purity), approximately 3–8 mm in length, serves as the tip. The tip is held in place by a custom-machined mechanical holder (Figure 2b). A steel AFM/STM 10 mm diameter sample disc (TedPella 16207), coated with a 100 nm-thick layer of Au through thermal or e-beam evaporation serves as the substrate (Figure 2c). Both the tip and substrate are mounted on small permanent magnets, which provide sufficient mechanical stability to prevent lateral (*x*–*y*) motion or wobbling during vertical (*z*-axis) actuation. Figure 2d shows a zoomed-in photo of a hand-cut tip driven into contact with a Au-coated substrate. To measure the conductance, a voltage is applied to the tip (or the substrate) using a data acquisition card and current from the substrate (or the tip) is measured using the transimpedance (current) amplifier. All wiring is made with coaxial cables to shield the signals from AC noise.

**Figure 2:**
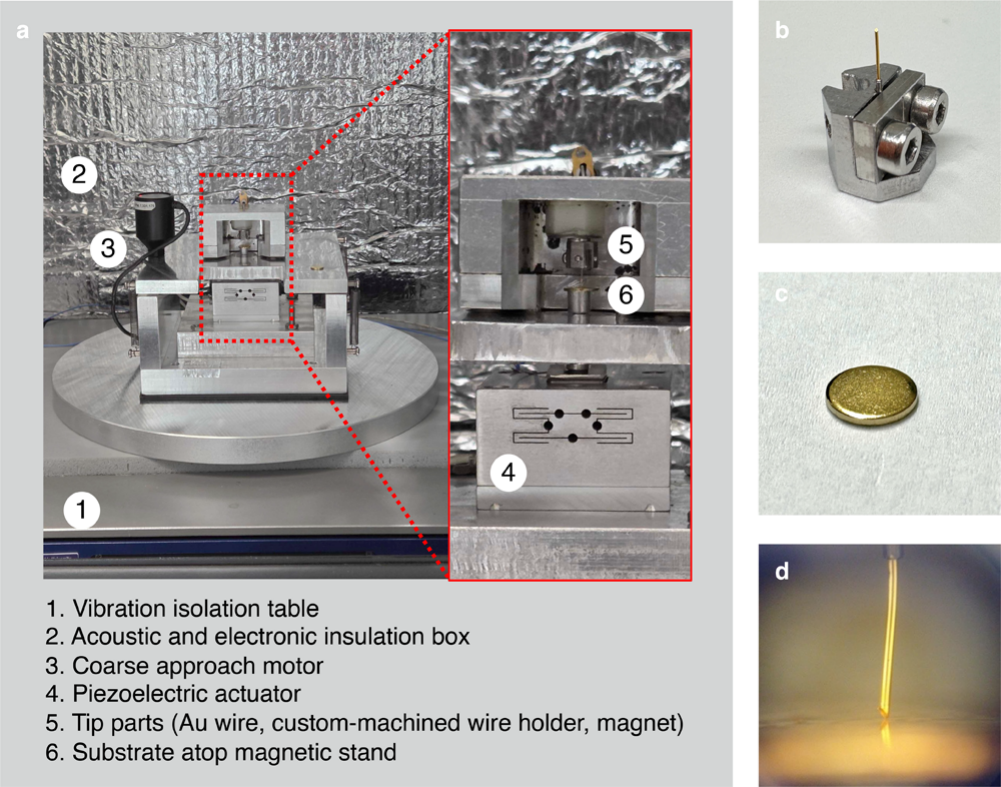
a) STM-BJ experimental setup on a vibration isolation table in an acoustic and electronic isolation box. The tip is magnetically secured to an upper platform that can be raised and lowered by a coarse-approach motor with sub-μm-scale steps. The substrate is positioned on a magnetic stand coupled to a piezoelectric actuator for sub-angstrom-scale steps. b) Custom-machined tip holder. c) Substrate made from a steel disc coated with 100 nm of Au. d) Close-up image of a Au wire tip driven into contact with a substrate.

Relative motion of the substrate and tip along the *z*-axis is achieved through a combination of two mechanisms: a coarse-approach motor provides μm-scale movements for the initial approach while setting up the instrument for an experiment, and a piezoelectric actuator enables sub-angstrom-scale precision movement during the measurement. In the setup shown in Figure 2, the coarse-approach motor raises and lowers a steel platform to which the tip is secured, while the piezo moves the substrate. Note that many variations of this design are possible; the piezo can be positioned to move either the tip or the substrate. The coarse-approach motor is controlled through software provided by the manufacturer, while the piezo actuator moves in response to a voltage applied through the STM-BJ electronics. The piezo movement is initially calibrated using interferometry. After calibrating a reference piezo, piezos in other setups can be calibrated relative to the first by matching the plateau length of a known molecular junction.

A circuit diagram of a sample STM-BJ setup is shown in Figure 3. The tip and substrate are moved in and out of contact with sub-angstrom-scale control using a piezoelectric actuator. Experimental parameters including piezo motion (driven by voltage V_P_) and the applied junction bias V_A_ are set within an experimental user interface and a 24-bit digital to analog converter outputs these voltages to the instrument. Current I_in_ flows across the junction electrodes in response to V_A_ and a current amplifier converts this to a voltage signal V_K_. A gain is set within the user interface and is communicated directly to the current amplifier, which determines the detection sensitivity (V_K_/I_in_ = 10^Gain^). To prevent overload of the amplifier, a series resistor R_series_ (typically 10 Ω - 1 MΩ) is used to limit current when electrodes are in contact. The resistor also attenuates voltage in regimes of low resistance when the junction resistance is smaller than the series resistance. This enables measuring a larger dynamic conductance range without having to change the amplifier gain. Care should be taken to tailor the value of R_series_ to the conductance of junctions being measured (smaller R_series_ for higher conductance), to avoid significant proportions of the applied bias from dropping across R_series_ rather than across the junction. The analog to digital converter collects V_K_ from the current amplifier and V_M_, the actual junction bias measured after the series resistor, and communicates this data to the user interface. Conductance is then calculated as G = I_in_/V_M_. Note that a voltage follower might be necessary depending on the input impedance of the actual data acquisition cards used in the setup. If the input impedance is low (e.g., 1 MΩ), the measured V_M_ will be less than the true junction bias; a voltage follower prevents a significant discrepancy.

**Figure 3:**
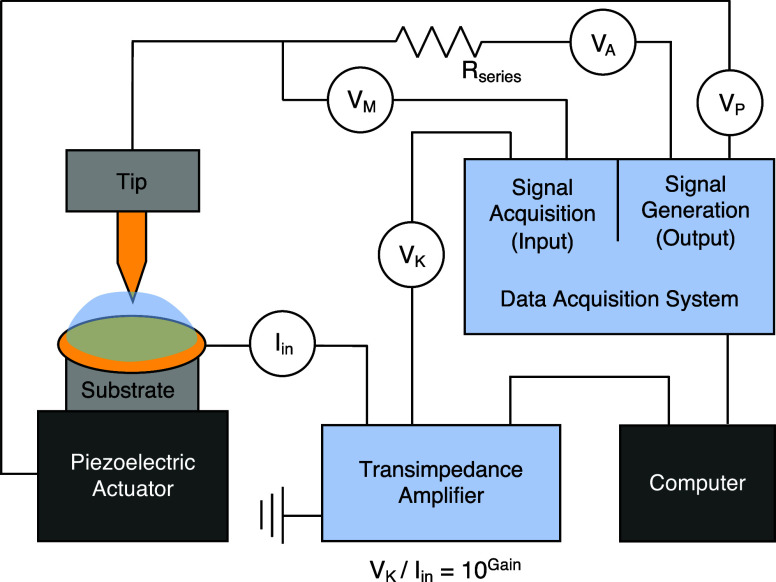
STM-BJ diagram. A Au tip and substrate form the junction electrodes. A voltage V_P_ is applied to the piezoelectric actuator to control relative motion of the tip and substrate. A junction bias of V_A_ is applied across the electrodes with a resistor R_series_ in series. The actual junction bias V_M_ is recorded. Current I_in_ across the electrodes is converted to a voltage V_K_ by the transimpedance amplifier. All voltages are measured using a National Instruments data acquisition card.

The STM-BJ instrument is exquisitely sensitive to external vibrations, including acoustic noise. Setups should be constructed on a vibration-isolated surface (e.g., an air table, active vibration isolation, or suspended using long bungees) and either acoustically shielded or located in a quiet room. The mechanical design integrating the tip, substrate, magnets, piezo, and stepper motor is flexible and can be implemented in a number of ways. However, it is crucial that the tip and substrate are vibrationally isolated as a single rigid assembly (for example, connected to a common large metal block), ensuring that any relative motion arises solely from the controlled actuation of the piezo or coarse-approach motor. Instrument cables should not be bundled or constrained, as overly stiff or tensioned cables can transmit vibrations that can be seen in the conductance traces.

Measurements are also highly sensitive to competing electronic noise, particularly from nearby AC power sources and electronic devices. Proper grounding is essential and should be achieved using high quality BNC coaxial cables with intact shielding and secure, tight connections. The shield must remain continuous throughout the setup and should be extended to any exposed metal components that could act as antennas. To prevent ground loops, all ground shields of the coaxial cables collecting data for the measurement should be connected to the same ground. It may be necessary to enclose the setup in a Faraday cage or similar shielding box to further protect the measurement from unwanted electrical interference. Electronic noise can be further minimized by physically spacing AC-carrying cords and devices away from DC data lines. Mechanical vibrations, acoustic noise, and AC pickup all result in easily recognizable artifacts in conductance traces, shown in Part [Sec sec4].

## 3 Data Collection

STM-BJ data is typically collected with an automated experimental procedure implemented in instrument-control software (e.g., Igor Pro). Once parameters (e.g., bias voltage, tip–substrate displacement distance) are set, the measurement can be run with minimal manual intervention. However, the measurement should be actively monitored, as some errors require pausing the experiment for parameter adjustments or mechanical troubleshooting. During data acquisition, the tip and substrate are repeatedly driven in and out of contact. During these make-and-break cycles, a bias is applied across the junction, the current is continuously recorded, and conductance is calculated in real time. For each cycle, conductance is plotted against the relative tip–substrate displacement, and these traces can be monitored to assess data quality and experiment stability. In this section we describe the preparation of the tip and substrate for experiment, the interpretation of conductance-displacement traces with recommended troubleshooting steps, and the experimental parameters necessary for experiment automation.

### 3.1 Tip and Substrate Preparation

Au is the most commonly used electrode material for STM-BJ experiments, as it exhibits quantized conductance steps in integer multiples of 1 G_0_ and is resistant to oxidation under ambient conditions. Other conductive metals such as Pt/Ir have also been used for tips.^81,82^ When paired with an Au substrate, a thin layer of Au coats the Pt/Ir tip apex, such that the experiment still effectively probes Au–Au point contacts. Here we provide instructions for the preparation of Au electrodes as this is the most widely adopted configuration.

The STM tip is prepared by cutting a segment of 250 μm Au wire at an acute angle using clean, sharp wire cutters. Under a microscope, the tip should appear pointed but not overly tapered or needle-like. Excessively thin tips are prone to bending or deformation after repeated contact. With experience, it is not necessary to check the tip under a microscope each time. Au-coated substrates can be cleaned using a UV-ozone or plasma cleaner, which removes organic contaminants and ensures a clean surface for junction formation. Before any experiment, it is essential to verify the integrity of the tip and confirm the instrument is functioning properly by collecting 1000 conductance–displacement traces of clean Au–Au contacts at 100 mV bias. These preliminary measurements ensure a functioning and stable tip, confirm a clean tip and substrate surface, and identify any sources of mechanical or electrical noise before a sample is added. Troubleshooting is far simpler without added molecular solutions, so any issues should be resolved at this stage.

### 3.2 Conductance-Displacement Traces

To begin a measurement, the tip and substrate are brought together by the piezoelectric actuator until electrical contact is established, as indicated by a sufficiently high conductance reading (e.g., ≫5 *G*_0_). They are then pulled apart while continuously recording the current, from which conductance is calculated and displayed as a function of tip–substrate displacement. As the contact narrows from bulk to a few atoms, the conductance decreases in quantized steps, close to integer multiples of the conductance quantum *G*_0_ = 2*e* ^2^/*h*. A short plateau at 1 *G*_0_ corresponds to a single-atom Au contact. Upon breaking this contact, the conductance drops sharply by several orders of magnitude to the instrument’s noise floor, the level of which varies depending on the gain of the amplifier, the amplifier bandwidth at the measurement speed, the bias applied, and the extent of shielding from environmental noise. Conductance-displacement traces that show sharp, well-defined plateaus at or near 1 *G*_0_ followed by a clean break are strong indicators of a well-prepared tip and clean substrate. An example trace showing these features is provided in Figure 4a. After collecting 1000 Au–Au traces, the system is considered ready for molecular measurements.

**Figure 4:**
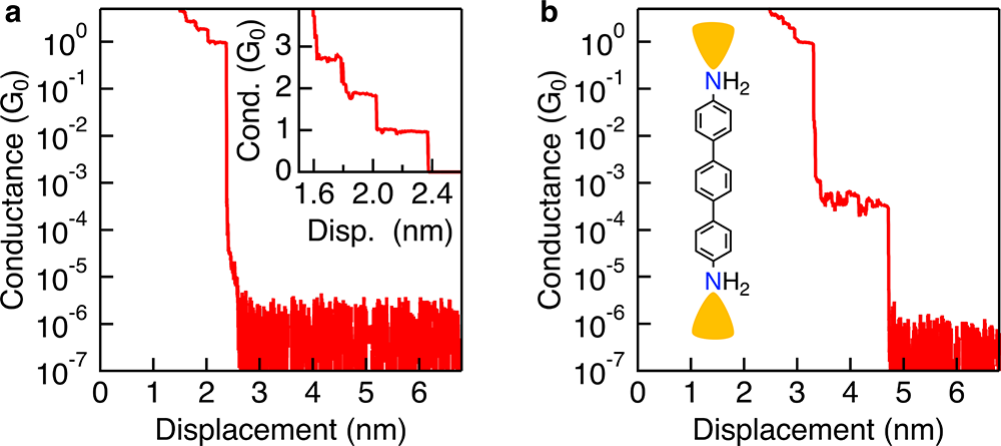
a) Sample conductance-displacement trace from a measurement of clean Au–Au contacts at 100 mV bias. At 0 nm displacement, the Au–Au contact conductance is greater than 5 *G*_0_. A plateau corresponding to single atom contact at 1 *G*_0_ breaks cleanly to instrument noise at a displacement of approximately 2.4 nm. Inset: linear scale graph showing integer multiples of *G*_0_ as Au–Au contact narrows from a 3- to 1-atom cross-section. b) Sample trace from measurement of 100 μM **pTDA** in TCB at 250 mV bias. Note that the noise floor is lower as compared with (a) as the bias is higher. Inset: **pTDA** structure coordinated to Au electrodes via N lone pairs.

STM-BJ measurements are typically performed in solution at room temperature. Samples are prepared in nonpolar, lower-volatility solvents^83^ such as 1,2,4-trichlorobenzene (TCB) or tetradecane (TD). Nonpolar solvents minimize background conduction through the solution while high boiling points ensure that in the time it takes to collect several thousand traces (at roughly 1–2 s/trace), the small solution volume (less than 100 μL) will not be reduced significantly by evaporation. Measurements in lower-boiling solvents can be performed by building a fluid cell around the substrate using a larger volume of solution, though this complicates the measurement and can lead to cross-contaminations from the fluid cell. Polar solvents (e.g., propylene carbonate) may be used, but require additional considerations discussed in Part [Sec sec6], such as insulating the tip to avoid background conduction through the solution. Measurements can also be performed in solvent-free conditions by dip-coating the substrate in an aqueous or volatile solvent solution containing the analyte molecule, then allowing the solvent to evaporate prior to measurement.^84–86^ This technique avoids some solvent-related constraints related to polarity, solubility, and evaporation during the measurement.

When performing solution-phase measurements, concentrations are typically 50–150 μM. In this range, a high percentage of traces show molecular junction formation, and single-molecule junctions are more probable than junctions bridged by two or more molecules simultaneously. Due to the sensitivity of the STM-BJ technique, solvents and samples must be of high purity (typically >98%). Impurities can interfere with junction formation, contaminate the electrodes, and introduce false peaks. A few drops (20–50 μL) of solution are deposited directly onto the substrate.

When a sample is added and a conducting molecule bridges the electrodes, an additional plateau appears after breaking the Au–Au point contact between 1 *G*_0_ and the instrument noise floor (Figure 4b). For the purpose of this tutorial, example measurements presented in this section contain **pTDA**, a molecule that has been extensively studied in STM-BJ experiments and exhibits a clear molecular conductance signature. Junctions with **pTDA** form via Au–N coordination, where the amine lone pair binds to the Au electrodes through a donor–acceptor bond.^55,56^ Amine linkers yield some of the most reproducible STM-BJ conductance signatures, along with pyridine and methylthio (-SMe) groups. More detailed information on the selection and performances of various linker groups compatible with STM-BJ measurements can be found in other works.^36,87–90^

The length of this molecular plateau correlates approximately with the length of the molecule as measured between the anchoring groups.^91^ Upon breaking the Au–Au atomic contact, the electrode geometries relax in a process known as electrode “snapback,” which results in the measured displacement underestimating the electrode separation, and a mismatch between plateau lengths and true molecular lengths.^91,92^ The snapback distance varies depending on the electrode geometry and experimental conditions such as temperature^93^ or solvent environment.^94^ Therefore, it is incorrect to assume or apply a fixed snapback value (for instance, 0.5 nm) across all measurements. The extent of the snapback also varies from trace to trace, which contributes to the variation in plateau lengths observed in histogram analysis.

Conductance plateaus vary in shape and duration due to changes in tip geometry (including snapback), molecule conformation, and Au-linker binding configurations.^95,96^ This is evident in both the fluctuations within a single plateau and in comparing features between individual traces. Conductance measurements are typically made at a data acquisition rate of 40–100 kHz, and any fluctuations in the measured current are due to slower processes. Changes in conductance that occur at rates faster than 1 μs (e.g., changes resulting from bond rotations) cannot be directly captured in the time domain using standard current amplifiers, considering their bandwidths at these acquisition rates. Only the influence of these effects on the most probable conductance of a junction may be established.^97^ To draw conclusions from single-molecule junction measurements, thousands of individual traces must thus be collected and analyzed; ensemble averaging can provide representative conductance values and structure–function relationships.

### 3.3 Diagnosing Errors from Traces

As mentioned above, 1000 preliminary traces with clean Au contacts must be collected prior to introducing a sample solution. These traces can be visually assessed to quickly identify and resolve problems related to the contacts, electronics, or experimental parameters. Here we provide a few common features of problematic traces and troubleshooting suggestions.

Poor tip geometry or a dirty tip/substrate are the most common causes of many irregularities in traces. Traces that lack a clearly defined *G*_0_ plateau (Figure 5a) and instead show a gradual conductance drop in this region may indicate a poorly formed tip. Another indicator of a weak or unstable tip may be Au–Au contact failing to break or requiring a long tip–substrate displacement (>5 nm) for conductance to drop to the instrument noise floor (Figure 5b). The Au surfaces at room temperature are dynamic; the geometries of both the tip and substrate are reformed each time they make contact, so these errors are sometimes resolved over time as more data is collected. However, the persistence of these problems over a few hundred traces would indicate that the bulk structure of the tip is not suitable or unstable. Therefore, if traces are highly irregular, fail to break to the instrument noise floor, or repeatedly show noisy/sloped transitions, a simple first troubleshooting step is to recut the tip. This often resolves issues related to the shape of the Au contacts. The Au surfaces can also be reshaped by pausing the automated experiment and using the piezo control to manually move the tip a few hundred nanometers in and out of contact several times. Similarly, traces may show an abrupt drop from many-atom contacts (>10 *G*_0_) to instrument noise without a defined 1 *G*_0_ step (Figure 5c). This indicates a blunt, nontapered tip shape, and can also be resolved by recutting or by using the piezo control to manually reshape.

**Figure 5:**
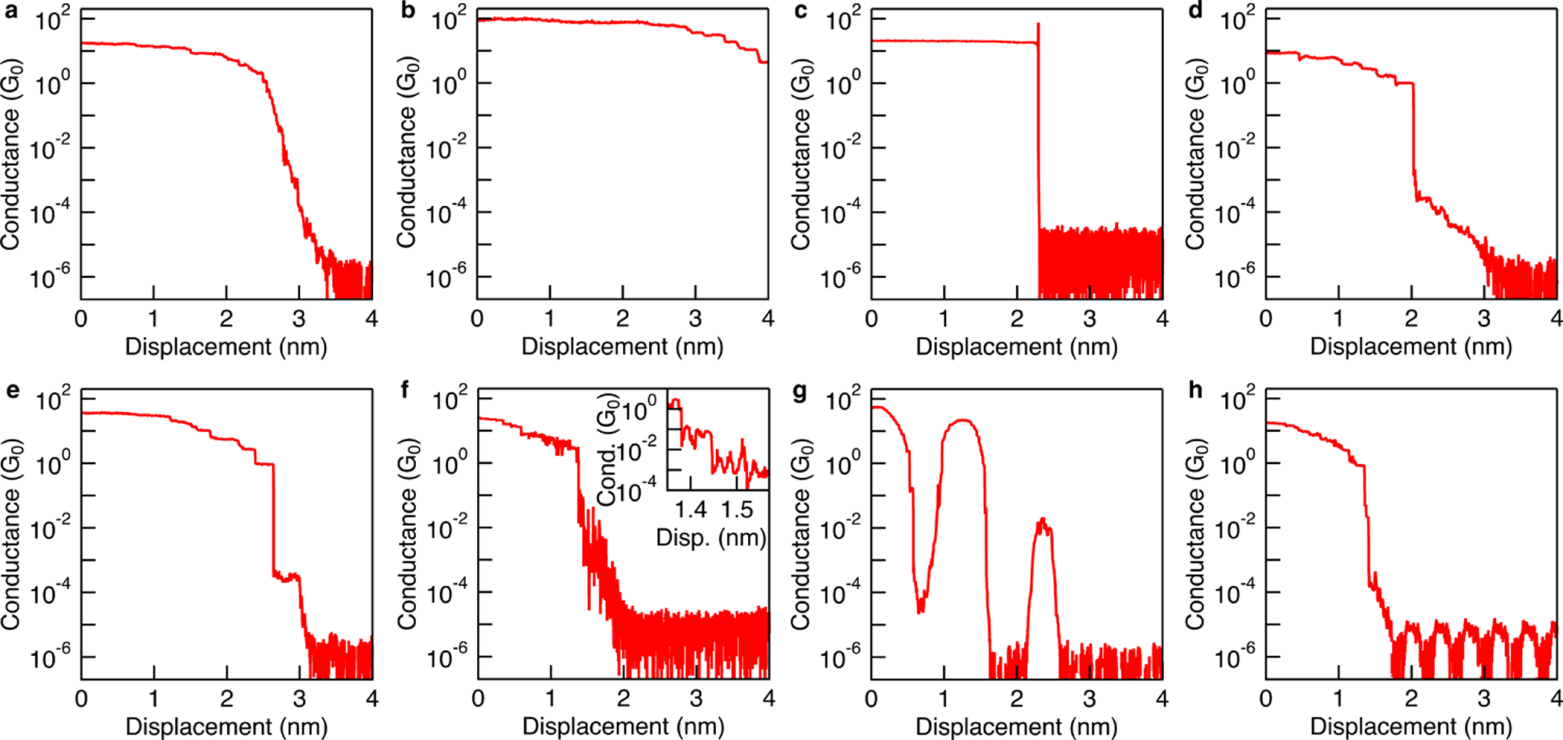
Common problems in conductance traces, such as a) sloping or poorly defined *G*_0_ plateaus, b) junctions that fail to break or require long displacements to break within the preprogrammed excursion, c) abrupt drops to noise from many-atom contact without establishing single-atom contact (the spike at 2.3 nm is due to the Butterworth antialias filter in the data acquisition card which is prominent when there are large changes in conductance), d) sloping or tapering at low conductance values near the instrument noise floor, e) sub-*G*_0_ conductance features in clean Au–Au measurement before sample is present, f) acoustic noise at around 700 Hz (inset: expanded view showing conductance fluctuations), g) mechanical vibrations at 20 Hz, in this case caused by a vibration isolation malfunction, or h) AC electrical noise resulting from ground loops.

Custom algorithms in the data acquisition code can be introduced to identify and discard occasional traces that do not exhibit clear features at 1, 2, or 3 G_0_ (indicating no Au–Au point contacts are formed) or those which do not break to the instrument noise floor, as these are considered incomplete and are excluded from histogram analyses. If conductance traces show gradual, noisy declines to the instrument noise floor rather sharp drops (Figure 5d), or if sub-*G*_0_ plateaus appear before introducing the sample solution (Figure 5e), the tip or substrate may be contaminated and both should be replaced.

One-dimensional (1D) conductance histograms (discussed in detail in Part [Sec sec5]) can be set to automatically generate for every 100 traces recorded. Monitoring these histograms alongside the individual conductance traces helps assess data quality in real time, such as the consistency of the *G*_0_ peak and the extent of any contamination present. Errors can disappear as the tip geometry evolves; if noisy traces are very infrequent and do not impact the appearance of the histograms, the experiment can proceed.

The initial set of Au–Au measurements also serves as a valuable diagnostic tool for mechanical issues. As shown in Figure 5f-h, acoustic, mechanical, and AC electrical noise sources produce identifiable artifacts in conductance traces. Acoustic noise (Figure 5f) results in high-frequency oscillations and can arise from poor sound insulation or sudden loud sounds (e.g., talking, doors slamming). Mechanical vibrations (Figure 5g) result in lower frequency, irregular oscillations of varying amplitude, often caused by inadequate vibrational isolation, loose components, or imperfect physical contact between parts and magnets, to name a few. These are easiest to identify when using a pulling rate of 5 nm/s or slower. AC electrical noise from nearby sources (Figure 5h) appears as highly periodic oscillations at the mains frequency (50 Hz in Europe, India, China, or 60 Hz in the USA, for instance) and often results from improper grounding. Persistent AC noise can be rectified with an electronically insulating Faraday cage around the instrument, and by removing any ground loops in the instrument.

Another potential issue arises when the electrical contact near the tip and substrate is interrupted; for example, when solvent or analyte from a previous experiment has created an insulating layer between the substrate and its holder or at the tip holder. This can result in a lower applied bias and difficulty achieving high-conductance tip–substrate contact. Notably, small additional resistances in the circuit will result in the 1 *G*_0_ peak being shifted to lower conductance (e.g., 0.9 *G*_0_). It is therefore important to keep all instrument parts solvent free and ensure that all points of electrical contact are kept clean and dry.

### 3.4 Experimental Parameters

Numerous parameters can be optimized to ensure data are accurately recorded and contact forms and breaks reproducibly. A list of parameters is provided in Table 1, along with the default values used in our lab. All parameters can be adjusted to fit the needs of different data acquisition systems and experimental setups. The **acquisition rate** determines how frequently data points are measured, which in turn sets the resolution of conductance traces. This rate depends on the analog to digital converters used in the instrument. In our lab, the maximum is 200,000 samples/s using the National Instrument PXI-4461 card. However, the rate must be chosen to ensure that it is within the bandwidth of the current amplifier at the specific gain setting used. The **amplifier gain** sets the range of currents that can be measured. A gain of 1 μA/V is sufficient to measure molecules with a conductance above 10^–6^ *G*_0_. Measuring lower conducting molecules requires a higher gain (0.1 μA/V) and slower pulling speeds.

**Table 1:** Key Experimental Parameters for Automated STM-BJ Measurements^a^

Parameter	Default Setting
Acquisition rate	40000 samples/s
Amplifier gain	1 μA/V
Pull speed	20 nm/s
Excursion	5 nm
Contact threshold	5 *G*_*0*_
Contact step size	0.5 nm
Deep indentation interval	50 traces
Deep indentation approach	30 nm
Deep indentation retraction	–40 nm

a Typical default values used in our lab are provided. Data acquisition parameters define how data signals are recorded, while motion control parameters (pull speed, excursion, contact threshold, contact step size) dictate the tip-substrate movement in each trace. Deep indentation parameters dictate the periodic, controlled reformation of the Au contacts to maintain stable tip geometry during extended data collection and to ensure that a large set of electrode geometries are sampled during the measurements.

The remaining parameters relate to the automation of the tip–substrate relative motion. **Pull speed** defines how quickly the piezo withdraws the substrate from the tip (or the tip from the substrate). The pull speed can be optimized for each individual setup and will depend on the specifications of the piezo, current amplifier, and data acquisition system. Pulling too slowly may allow more vibrations to impact the data. Our lab has used pull speeds ranging from 1 to 750 nm/s, though the data quality is reduced at high speeds.^98^ The **excursion** is the set displacement distance. If traces are consistently breaking toward the end of the total displacement distance, or if long molecules are being measured, it is advisable to increase the excursion to ensure all junctions break and instrument noise is detected before the next trace starts. For example, if a 1 nm long molecule is measured, a good starting excursion is 7 nm. For much shorter molecules, a 5 nm excursion is sufficient. Increasing the excursion can also help maintain a usable tip condition when measuring analytes capable of strongly binding and distorting the electrode surfaces (e.g., thiols). The **contact threshold** defines the conductance when the tip and substrate are brought into contact to indicate that separation of the contacts can be initiated. **Contact step size** defines the incremental distance by which the electrodes are brought together to establish contact. In our lab, the approach trace is not recorded and the substrate moves toward the tip in large steps to decrease data acquisition times. Reducing the step size allows finer control and reduces the risk of creating very large contacts at the start of the trace.

Periodically throughout the measurement, the tip and substrate are brought into extended contact to reshape the electrodes more substantially than during a standard trace. The **deep indentation interval** determines how often this event occurs; we typically set deep indentation to occur once per 50 traces collected. The extent of this contact is defined by the **deep indentation approach** and **deep indentation retraction** distances. This periodic reformation of the Au contact ensures that the tip and substrate surfaces are changed considerably throughout the measurement to sample different contact geometries for molecular junctions.

## 4 Data Analysis: Conductance Histograms

Once several thousand conductance–displacement traces have been recorded, the resulting data can be analyzed statistically without selecting or filtering individual traces. In typical STM-BJ experiments, the molecular junction formation or “pickup” rate is relatively high; for molecules that are 1 nm or longer, this rate is greater than 90% as long as solution concentrations are at least 10 μM. As a result, all consecutively recorded traces should be included in the analysis, regardless of whether a molecule appears to be present in each individual trace. It is unnecessary to filter out traces that do not show a molecular conductance plateau and instead show a clean drop from 1 *G*_0_ to the noise floor, as these traces do not significantly alter the histograms. Filtering out data that have sloped plateaus or other unexpected features may introduce selection bias and obscure important physical or chemical insights, such as multiple binding geometries or low-probability events.

The most common methods of data presentation are one-dimensional (1D) conductance histograms and two-dimensional (2D) conductance–displacement histograms. Individual traces are smoothed prior to generating histograms. In our analysis, we apply a box smoothing (level 11) to all traces. In a 1D histogram, conductance bins are generated on a logarithmic scale. We use 100 bins per decade and accumulate all conductance data in these bins. These 1D histograms are then plotted as counts per trace versus conductance. When comparing the 1D histograms in Figure 6 with and without a molecule present, it is important to note that the noise floor peak shifts to slightly lower conductance in Figure 6c, e. This is a result of increasing the bias from 100 mV to collect the initial Au–Au traces, to 250 mV for the molecular measurement. Since the noise floor is dictated by the smallest current that can be measured with the current amplifier, a higher bias yields higher currents and thus conductance peaks are more distinguishable from the noise floor.

**Figure 6:**
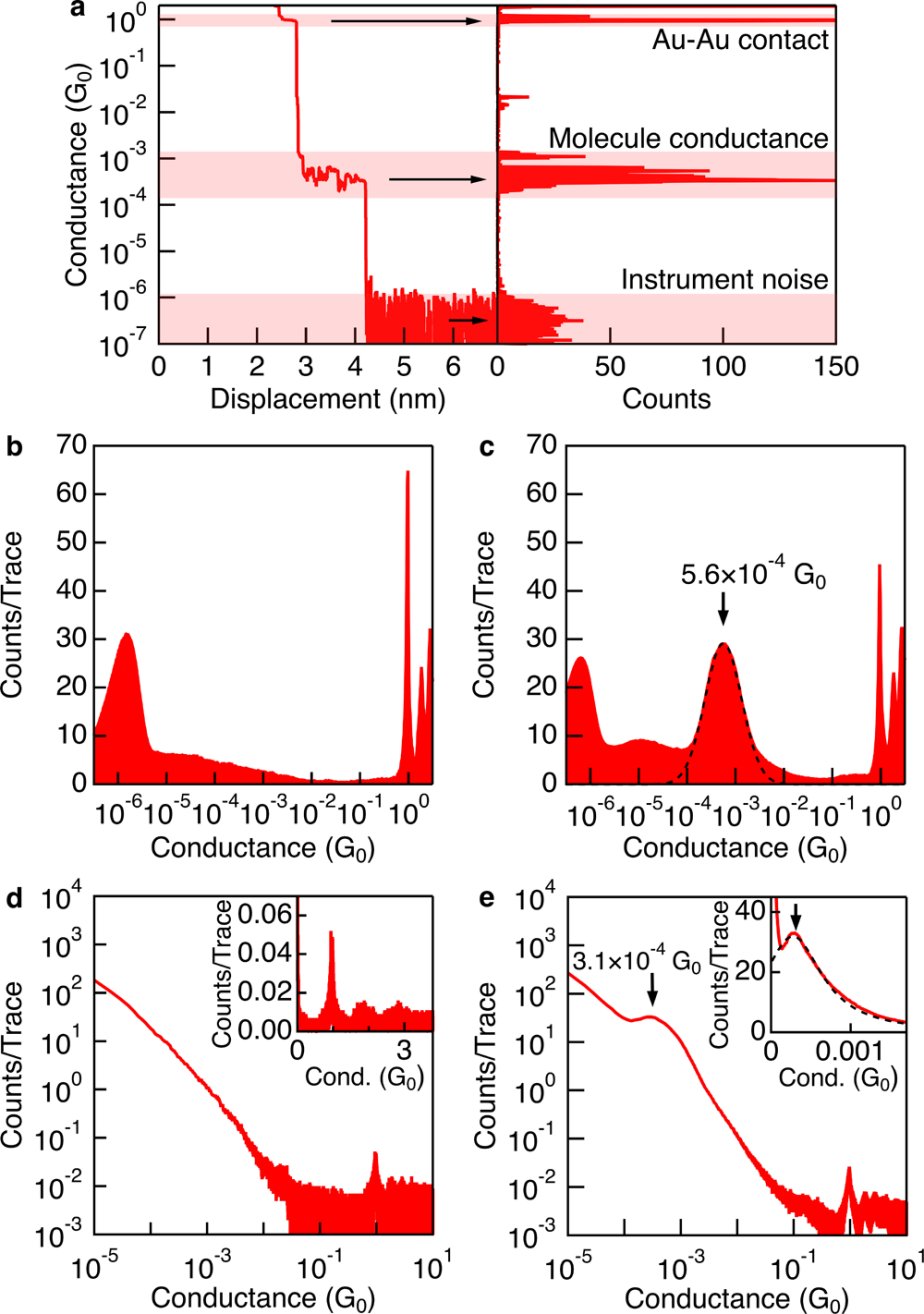
a) Single conductance trace measured with **pTDA** at 250 mV bias along with a logarithmically binned (100 bins/decade) 1D histogram generated from the trace, highlighting the key regions of the histogram. b) 1D histogram generated from 1000 initial Au–Au traces collected at 100 mV bias prior to adding a sample, showing a well-defined 1 *G*_0_ peak and a broad feature at 10^–6^ *G*_0_ reflecting instrument noise. These peaks represent upper and lower bounds for molecular conductance to be observed at this bias. c) Logarithmically binned 1D histogram of 10000 **pTDA** conductance-displacement traces measured at 250 mV in TCB, showing a molecular conductance peak between the 1 *G*_0_ Au contact and 10^–6^ *G*_0_ instrument noise peaks. This peak is fit with a Gaussian to determine the most probable conductance. Instrument noise should not be mistaken for a molecular conductance feature, and is typically omitted from histograms for clarity. The instrument noise peak position shifts slightly from (b) to (c) due to the change in applied bias. d) Linearly binned 1D histogram generated from the 1000 Au–Au trace data set shown in (b) using a bin size of 10^–5^ shown on a log–log scale. Inset: Region around 1 *G*_0_ shown on a linear scale. e) Linearly binned 1D histogram generated from the 10000 trace **pTDA** data set shown in (c) using a bin size of 10^–5^. Inset: Region around the molecule peak shown on a linear scale. Molecular conductance is observed as a peak between 10^–4^ and 10^–3^ *G*_0_ and the peak position is determined using a Lorentzian fit to the data. Note that the conductance peak position is different when comparing linear and log binned histograms as indicated in the figure.^99^

In early STM-BJ literature, histograms were often constructed with linear binning. This provides high resolution at higher conductance values (e.g., for studying point contacts), but lower conductance features may be obscured and broad peaks would be seen simply as increased counts and not a clear peak. Logarithmic binning was introduced by Gonzalez et al. in 2006.^100^ They showed that the tunneling background in conductance traces—appearing as an increasing count at low conductance in linearly binned histograms—transformed into a constant background when logarithmic binning was applied. We also found that broad conductance features in linear histograms become well-defined peaks on a logarithmic scale, facilitating quantitative analysis by Gaussian fitting to extract characteristic conductance values of molecular junctions. Importantly, there is no single “correct” method for representing the data; rather, each approach emphasizes different aspects of the distribution. A clear understanding of the distinctions and limitations of linear versus logarithmic binning is therefore essential for accurate interpretation. Figures 6b-e provide comparisons of logarithmically binned and linearly binned 1D histograms generated from the same data sets. The **pTDA** conductance feature (Figure 6e) appears in a linearly binned histogram as a broad peak on a sloped background with an onset at approximately 10^–3^ *G*_0_. Logarithmic binning is now standard for reporting molecular conductance data, although the conductance values obtained from logarithmically binned data are skewed to higher values as shown in Figure 6.^100^

The histogram resolution is best if a minimum data set of 3000 traces is used. As shown in Figure 7, histograms constructed from 10 traces are highly jagged, and the conductance maximum observed in the 1D histogram does not necessarily correspond to the most probable conductance value. However, as additional traces are compiled—100, 1000, and 10000—a clear conductance distribution emerges. At high trace counts, the histogram peak between 10^–4^ and 10^–3^ G_0_ develops a well-defined Gaussian profile (Figure 7d), from which a statistically meaningful peak value can be extracted through a least-squares fit to the data.

**Figure 7:**
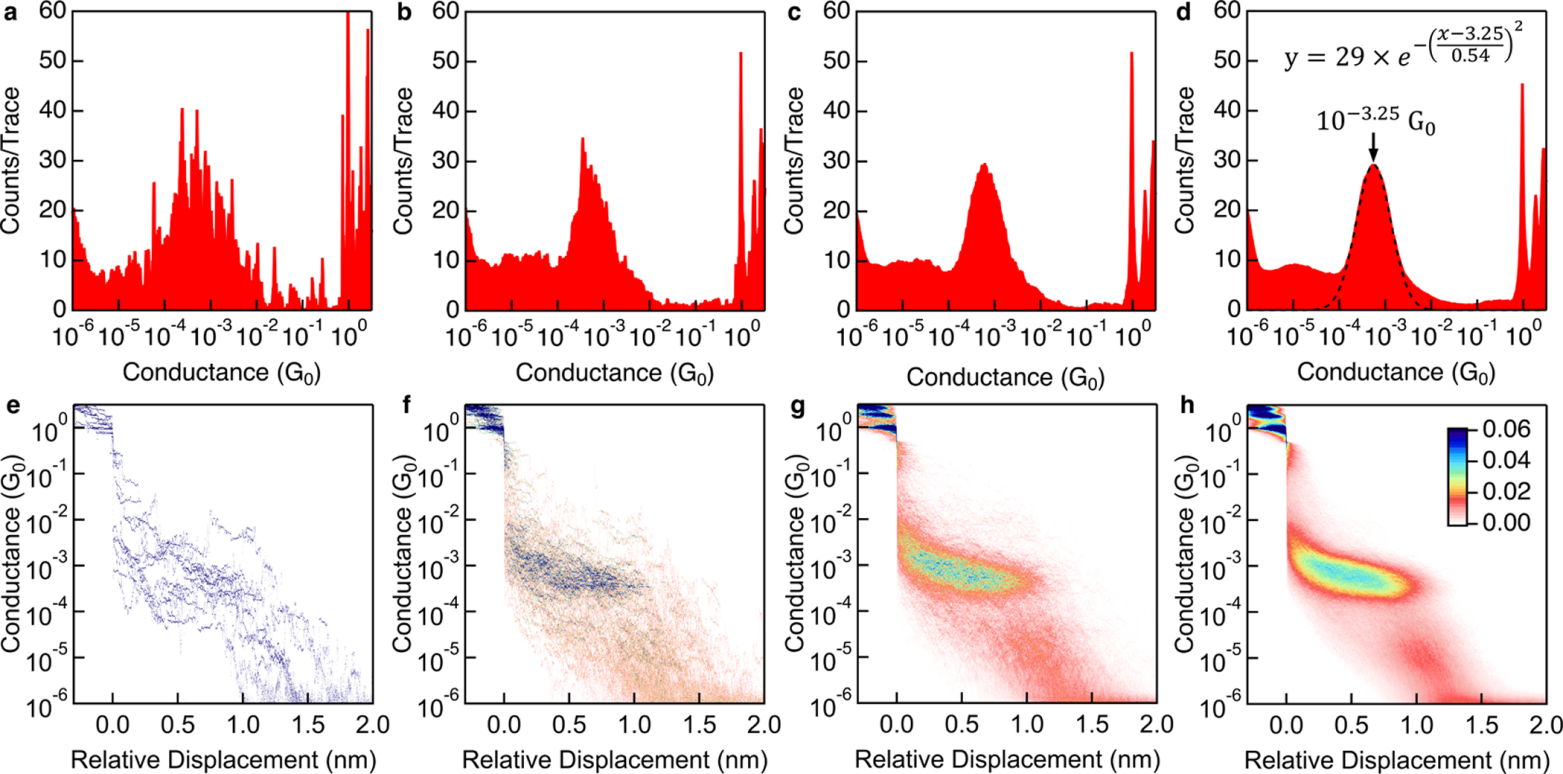
1D (a–d) and 2D (e–h) histograms generated from 10 (a, e), 100 (b, f), 1000 (c, g), and 10000 (d, h) consecutively collected conductance traces in a 100 μM solution of **pTDA** in TCB measured at 250 mV bias. The peak corresponding to instrument noise is typically cropped from these histograms for clarity. The color scale displayed in (h) represents counts per 2D bin and applies to all 2D histograms in this panel, normalized to the number of contributing traces. Note that a second peak around 10^–5^ *G*_0_ appears in the histograms with 10000 traces. This is due to a molecular π-stacked dimer as will be discussed further below.

When fitting the data, the following points must be taken into consideration:

(1) The baseline for the Gaussian fit should be constrained so the tails of the Gaussian approach zero.
(2) It is essential that the presence of the peak is unambiguous. It should be recognizable with or without a Gaussian fit overlaid in a 1D histogram (or an oval overlaid in a 2D histogram).
(3) The peak should be distinct from any noisy background current and should not overlap significantly with the instrument noise floor.
(4) Any background subtraction based on clean Au measurements tends to be inaccurate, as the electrode structure is changed once a sample is added.
(5) When presenting data, the *G*_0_ peak should be included in all histograms; the height and sharpness of this peak reflect the data quality.
(6) It is common practice to display a conductance range that excludes the instrument noise peak, to avoid readers mistaking this feature for molecular conductance. Though cropped from figures, the noise is *not* subtracted from the data itself.

In addition to 1D histograms, data can also be represented using two-dimensional (2D) conductance–displacement histograms where conductance is plotted on the *y*-axis (again using 100 logarithmic bins per decade) and displacement on the *x*-axis using linear bins, replicating the layout of individual conductance traces. The displacement for each trace is first set to zero at the location where the conductance crosses 0.31 (10^–0.5^) *G*_0_ and the traces are then binned into the 2D map using a color scale to indicate the frequency of occurrence of data points across all recorded traces.^91^ These histograms, shown in Figure 7e-h, can be thought of as an overlay of all conductance–displacement traces with the point where conductance drops below 1 *G*_0_ set to zero relative displacement. These 2D histograms reveal the most probable junction evolution pathways. Similar to 1D histograms, it is best practice to include the *G*_0_ feature in 2D histogram (at negative relative displacement). When comparing 2D histograms across multiple experiments, it is essential that the color scale be consistent and normalized with respect to the number of included traces. Furthermore, the feature in the 2D histogram should be clearly visible, and should not require an oval overlay to distinguish the feature from the noise floor.

Statistical processing of large data sets not only yields reproducible average conductance values and more accurate quantitative conclusions, but also enables the identification of lower probability junction events that may be entirely absent in smaller subsets of data. For example, the 2D histogram constructed from 10000 **pTDA** traces shown in Figure 7h shows a primary conductance plateau near 10^–3^ *G*_0_ and a secondary plateau near 10^–5^ *G*_0_. The secondary feature has been attributed to a dimer configuration, in which two terphenyl molecules interact via π–π stacking to form a parallel, cofacial dimer bridging the electrodes as we will discuss in Part [Sec sec6].^101^ This noncovalent geometry results in a weaker electronic coupling and therefore a lower conductance signature. Dimer junctions have a lower probability of formation compared to a single **pTDA** bridge, making it difficult to observe in histograms generated from fewer traces.

A subtle yet critical aspect of data analysis, and a common pitfall, is distinguishing between true molecular conductance peaks and contamination artifacts. While precautions should be taken to prevent contamination (e.g., maintaining clean electrode surfaces, tools, sample purity), false peaks may still arise. It is therefore essential to reproduce measurements using independently prepared solutions, tips, and substrates, before reporting results. Characterizing molecular conductance in multiple solvents is also recommended. If solvent molecules produce conductance artifacts, or if a solvent bottle has been contaminated, data may be consistently misinterpreted. Evidence of a clear peak from measurements in multiple solvents suggests the peak is intrinsic to the analyte molecule.

Additional sanity checks should be implemented while interpreting results. For example, plateau lengths may be compared with those expected for molecules of a similar length (as measured between linking groups). While plateau length is not a precise measure of molecular length, dramatic discrepancies are unlikely. Such comparisons should only be made between measurements in the same solvent, on the same instrument, since plateau lengths can vary based on the solvent and piezo calibration. More broadly, conductance values should be physically reasonable for a given molecular structure. Due to the sensitivity of the technique, all potential sources of contamination must be methodically considered before assigning peaks.

## 5 Variations on Basic Measurements

### 5.1 Polar Solvents and Coated Tips

As discussed in Part [Sec sec4], STM-BJ experiments are frequently performed in nonpolar, lower-volatility solvents. However, polar solvents are sometimes necessary, particularly for studying molecules that are poorly soluble in nonpolar media, biologically relevant molecules,^3,85,102,103^ charged species that carry counterions, or electrochemical measurements that involve redox processes or a supporting electrolyte. Polar low-volatility solvents such as propylene carbonate (PC) or dimethylformamide (DMF) can be used in these cases, requiring tip insulation as described below. Water can also be used, either in a fluid cell or by adding water drops throughout the measurement to compensate for evaporation. While most charged molecules require a polar solvent due to solubility constraints, there are exceptions when charged molecules with counterions can dissolve in TCB or bromonaphthalene; in these cases, measurements can proceed without tip modification.^26^

Immersed in a polar solvent, the entire surface area of the 250 μm-diameter wire tip is electrochemically active. A polar solvent can thus introduce increased background Faradaic and capacitive currents as shown in Figure 8a, especially at high bias, resulting in poor signal-to-noise ratios and potentially obscuring molecular conductance features Figure 8. To mitigate these effects, it is necessary to coat the STM tip with an insulating layer such as Apiezon wax,^104^ exposing only the apex of the tip. This reduces the surface area that would otherwise contribute to background conduction (Figure 8b). A measurement of 5000 traces of **pTDA** in PC is shown in Figure 8c for comparison. Slight differences in molecular conductance are observed when comparing measurements in different solvents; for instance, PC measurements typically result in slightly shorter plateau lengths than seen in TCB, likely due to a larger snapback.

**Figure 8:**
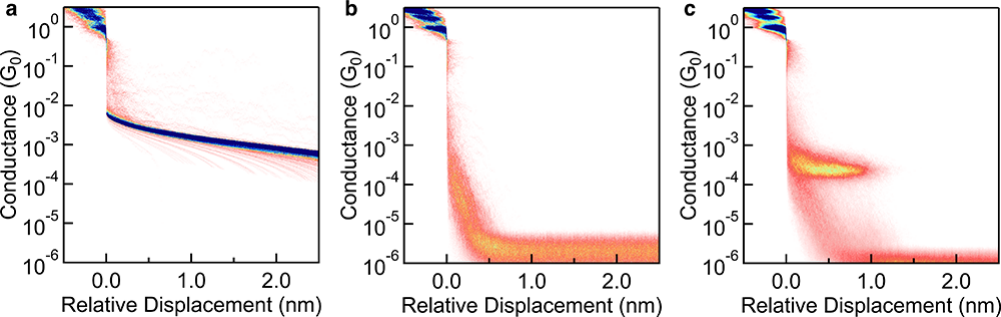
a) 2D histogram of 1000 traces measured in PC at 100 mV bias, using standard Au tip and substrate. Strong background currents are visible. b) 2D histogram of 1000 traces measured in PC at 100 mV bias, using an Apiezon wax-coated Au tip. c) 2D histogram of 5000 traces of 100 μM **pTDA** in PC at 250 mV bias using a coated tip.

Figure 9 presents the procedure used in our lab to coat Au tips with wax. A soldering iron heating element is fit with a custom-machined copper platform and immobilized by a clamp on a support stand. A small amount of wax is deposited on the plate and allowed to melt. For convenience of maneuvering the fragile tip, we insert a few mm of Au wire into a syringe needle with an inner diameter of 250 μm. The tip is then cut using sharp wire cutters and the shape of the tip is checked under a microscope. Holding the base of the needle, the Au tip is dipped into the wax and removed with a quick, upward flicking motion. Under a microscope, the tip should barely appear as a point protruding from the wax, as shown in Figure 9e. If too much of the Au apex is visible, as shown in Figure 9f, the coating is insufficient.

**Figure 9:**
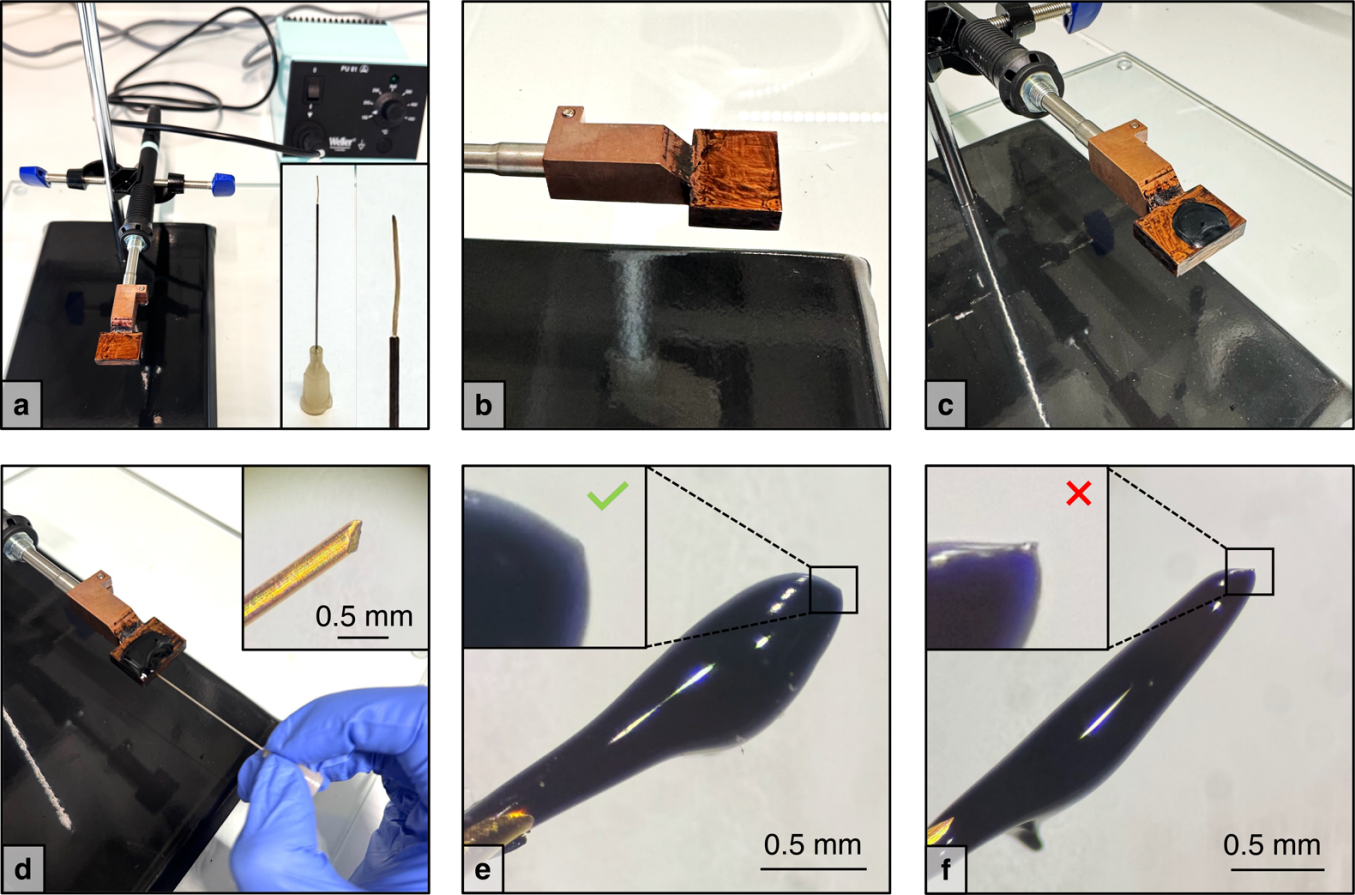
Procedure for coating tips for polar solvent measurements. a) Soldering iron with a copper platform fitted at the end. Inset: 250 μm Au wire positioned in a blunt syringe needle. b) Custom-machined Cu plate. c) Apiezon wax melted on plate. d) Tip is submerged and withdrawn from melted wax by hand. Inset: shape of hand-cut Au tip prior to coating. e) Well-coated tip as seen under microscope, with apex slightly visible. f) Insufficiently coated tip with excess Au exposed at apex.

Coated tips can introduce some experimental challenges. If wax is applied too heavily, tip–substrate contact can be difficult to establish, or difficult to reproducibly break due to the wax “sticking” to the substrate while the tip and substrate are pulled apart. If the wax is too thin, too much Au may be exposed and the challenges associated with polar solvents are not mitigated. It is again advisable to perform baseline Au–Au measurements to establish tip viability before introducing an analyte solution. For experiments with a coated tip, it might be necessary to add a drop of solvent for the baseline measurements.

### 5.2 Measuring Current–Voltage Characteristics

Collecting measurements in a polar solvent with a coated tip enables systematic control of bias polarity, allowing molecular conductance to be compared under positive and negative tip bias. These data can be used to construct current–voltage or *I–V* curves and observe rectification. During an experiment, the wax coating significantly reduces the exposed surface area of the tip electrode compared to the substrate. As a result, the bias produces a local electric field that is more concentrated at the tip apex and more dispersed at the substrate. When solvent dipoles reorient in response to the local field, the subsequent screening effect is more pronounced at the tip. In a standard experiment with a plain tip and nonpolar solvent (illustrated in Figure 10a), the electric field can be considered uniform, and reversing the bias simply inverts the field experienced by a bridging molecule. In contrast, in polar media with asymmetric electrode geometry, reversing bias polarity changes both the field direction and the distribution of the potential drop, and thus the field profile experienced by the molecule.

**Figure 10:**
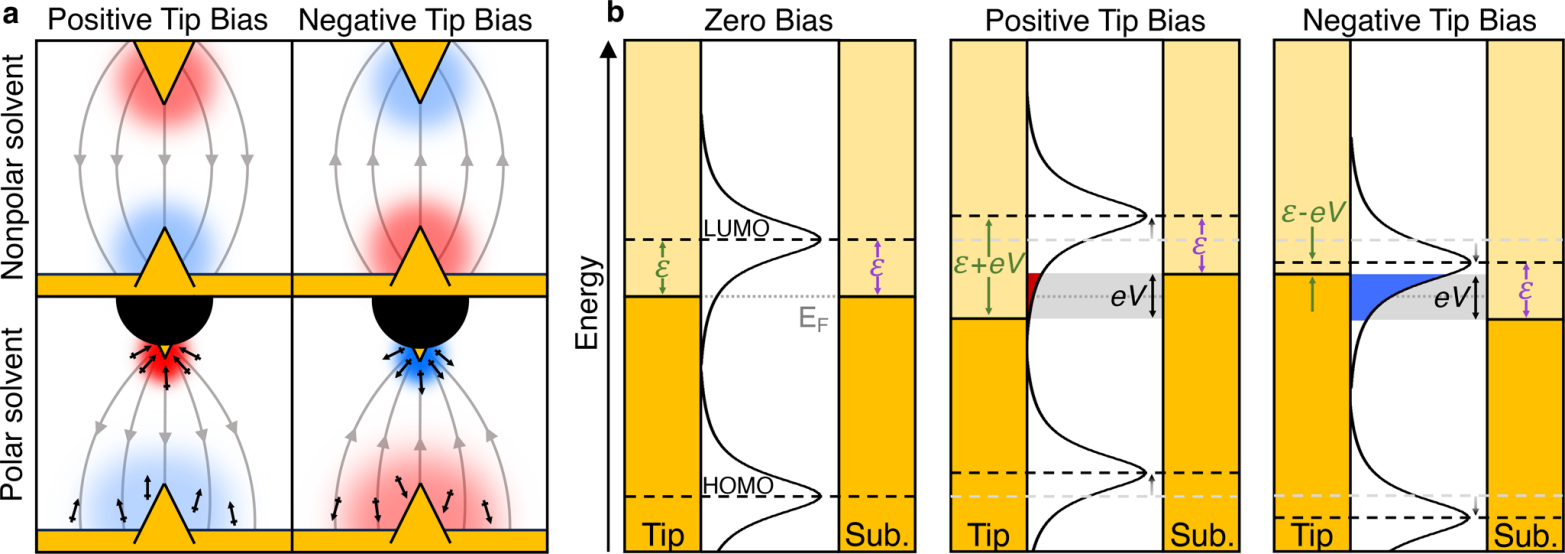
a) Schematic illustrating the effects of a polar solvent and coated tip on electric field profile. In a nonpolar solvent, bias reversal results in symmetric inversion of the field. A coated tip concentrates the field at the tip apex, while solvent dipoles screen the charge. Bias reversal inverts the field asymmetrically. b) Energy level diagram demonstrating shifts in molecular orbitals at positive and negative tip bias. The first panel shows alignment of LUMO resonance with energy *ε* relative to electrode Fermi level *E*_F_ at zero bias_._ The middle panel shows how a positive bias shifts the alignment of the junction Fermi level relative to the orbitals while the right panel shows the same for a negative tip bias. Note that the area under the transmission curve is very different for the positive (red) and negative (blue) tip bias.

The asymmetric field profile shifts molecular orbital resonances relative to the junction Fermi level. Specifically, orbitals shift in their alignment with the tip chemical potential, while the alignment with the substrate chemical potential remains rigid. This shift occurs in opposite directions for opposite bias polarity. As a result, orbital resonances can be probed at lower biases than in a system with a uniform field. Therefore, switching the bias polarity in a measurement can shift molecular conductance peaks, and in some cases alter the dominant conducting orbital. Importantly, the polarity dependence of molecular conductance means that rectification can be observed. These relationships along with an extensive series of rectification experiments in polar solvents have been discussed in Capozzi et al.^11^ Rectification is enhanced by the presence of a supporting electrolyte such as tetrabutylammonium hexafluorophosphate (TBAPF_6_) which enables the formation of a denser double layer around the tip. The experiment shown in Figure 11 was performed in a solution of 100 μM **pTDA** and 100 mM TBAPF_6_ in PC.

**Figure 11:**
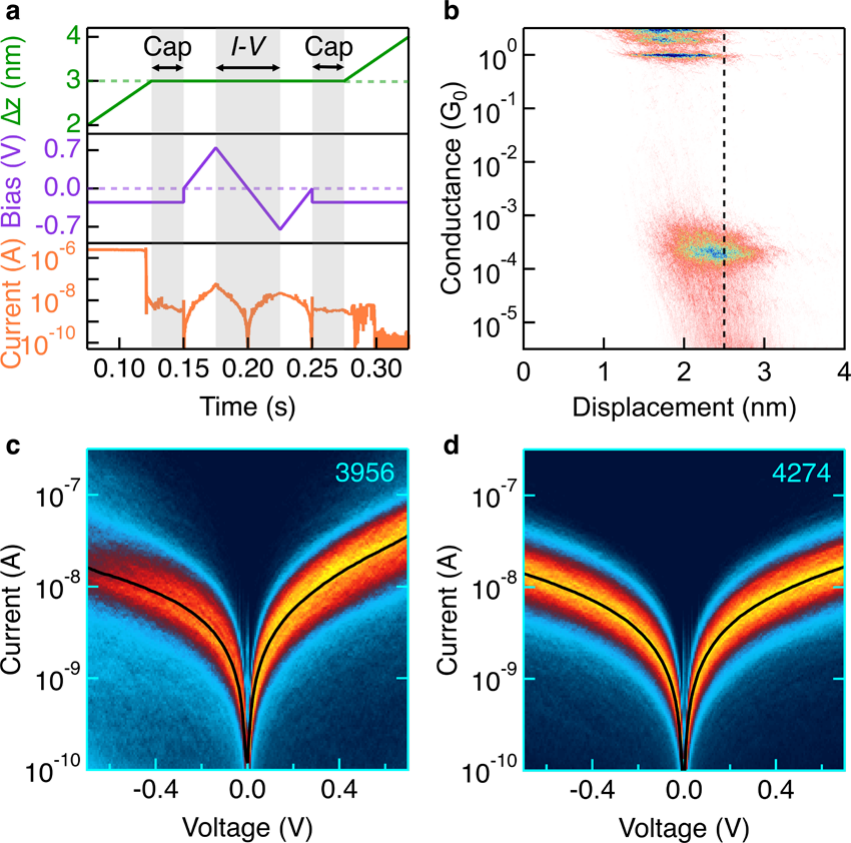
*I–V* measurements of 100 μM **pTDA** with 100 mM TBAPF_6_ in PC. a) Sample trace during *I–V* experiment. The tip–substrate distance (Δ*z*, green) is first increased until a junction has formed, then held constant over a bias ramp, then increased again to break the junction. The bias (purple) is first applied at a constant −0.25 V following the G_0_ break (start cap), then ramped from 0 V to +0.75 V, −0.75 V, and 0 V, then held constant at −0.25 V a second time (end-cap). The current (orange) during the start and end-cap is used to identify traces which hold a molecule. The current over the shaded central region of the bias ramp (from +0.75 V to −0.75 V) is used to generate *I–V* histograms. b) 2D unaligned histogram of 1000 **pTDA** standard conductance traces measured with a −0.25 V bias. The approximate onset of the conductance plateau (2.5 nm) is used to set the initial pull in an *I–V* experiment. c–d) *I–V* histograms from measurements of **pTDA** in PC (c) and TCB (d). The numbers in the upper right corners indicate the total number of traces selected based on start and end-cap conductance, which were used to construct these histograms.

To probe *I–V* characteristics, nonstandard conductance measurements must be performed. Rather than using a continuous pull speed to separate the tip and substrate, in these experiments we pause the piezo movement after a fixed displacement to hold a molecular junction and sweep the bias up and down.^105^ Once the sweep is complete, the electrode separation is resumed and the junction is broken. A sample trace showing tip–substrate relative displacement (Δ*z*), applied bias, and current response is shown in Figure 11a. Several new parameters and considerations must be made to implement this procedure.

First, in order to pause the piezo movement with a molecular junction intact, we must determine the displacement at which a junction is most likely to form. We first collect a minimum of 500 standard, continuous-pull traces and construct a 2D histogram without aligning the traces at the position of the G_0_ break. By analyzing this unaligned histogram, we can identify the displacement distance that will most likely form a molecular junction. Such a histogram is shown in Figure 11b, where it is clear that the highest number of junctions are present at a displacement of ∼ 2.5 nm, so we set this value as an “initial pull.”

Even with a carefully selected initial pull, the success rate of pausing the piezo movement at the precise moment a junction is formed is typically between 5 and 15% (if the rate appears much lower than 5%, the tip may need to be replaced). Unlike in standard conductance experiments, traces must be sorted and only those which trap a molecular junction during the voltage sweep should be used for analysis. It is therefore necessary to collect a higher total number of traces (at least 10000–20000) to ensure there are enough selected traces to construct meaningful *I–V* histograms. To identify which traces form junctions, we introduce “start-cap” and “end-cap” parameters. After the initial pull, the piezo motion is paused and the bias is held constant for 0.025 s (the start-cap) before the *I–V* sweep begins. If the average conductance in this window matches the expected molecular conductance at that bias, then we confirm a molecular junction is present and the trace should be used in analysis. Traces that do not show a molecular conductance signature in the start-cap are excluded from analysis. If many junctions rupture during the *I–V* sweep, a second constant-bias cap can be used at the end of the trace (the end-cap) to identify traces in which a junction is still intact after the sweep. Following the end-cap, tip–substrate separation is resumed to ensure the junction is broken and instrument noise is detected before beginning the next trace.

Once traces have been selected for analysis based on conductance signatures present in the start- and end-caps, they are compiled as current versus time histograms to show the full segment where the bias is swept from 0 V to +0.75 V to −0.75 to 0 V. These histograms enable visualizing how the junction responds to the bias. To make *I–V* histograms, current from the segment of the trace in which the bias is swept from +0.75 V to −0.75 V is plotted against the measured junction bias and collected as two-dimensional overlays. Each vertical slice of the *I–V* 2D histogram is then fit to a Gaussian to identify an average current at each voltage (the black curves in Figure 11c-d), and these values can be used to calculate the current rectification ratio. In Figure 11c the 2D *I–V* histogram obtained from a measurement conducted in PC shows a rectification ratio of ∼ 2.2 at ± 0.70 V bias. By contrast, Figure 11d illustrates a similar 2D *I–V* histogram from a measurement in TCB, in which the electric field profile is not modified upon reversing bias, and as a result no rectification is observed.

### 5.3 Flicker Noise Measurement

STM-BJ measurements are conducted at room temperature, making Au atoms near the apex of each electrode likely to undergo thermally activated rearrangements between metastable configurations. These fluctuations in Au atom positions influence the Au-molecule interface and the nature of molecule–electrode coupling, which can be probed by measuring flicker noise (1/f noise) as explained in detail in Adak et al. and Magyarkuti et al.^101,106^ Here, we outline the basic process for flicker noise measurement and data analysis, reproducing data from past publications.

Similar to an *I–V* measurement, the flicker noise measurement requires modification of the tip–substrate displacement and applied bias profiles. The piezo movement is paused at the point of junction formation, and the bias is held constant while conductance is recorded. The default acquisition rate of 40 kHz can be increased to 100 kHz to increase the data resolution within the hold segment and to ensure that the frequency dependence of the measured conductance follows a 1/f dependence. As described above in the *I–V* measurement section, an optimal initial pull distance must be identified prior to the measurement to increase the likelihood of junction formation. Junctions with molecular signatures are identified by determining the average conductance during the hold segment and ensuring that this is within the molecular conductance peak in the 1D histogram.

After data has been collected and traces containing junctions are selected, a discrete Fourier transform is performed on the constant-bias hold segment of each conductance trace, and the result is squared to determine the conductance noise power spectral density (PSD). A sample trace from a flicker noise measurement of **pTDA** is shown in Figure 12a. The corresponding PSD is shown in Figure 12b where a clear 1/f dependence is visible, the hall-mark for flicker noise. In the case of through-bond coupled junctions, this relationship is purely a result of Au atom rearrangements and exists irrespective of the bridging molecule structure. Without demonstrating this 1/f dependence, one cannot conclude that flicker noise is the origin of the observed conductance fluctuations. Furthermore, the sample PSD traces also clearly demonstrate the frequency range that can be analyzed in each experiment. Published data that do not include sample PSD traces are therefore not complete.

**Figure 12:**
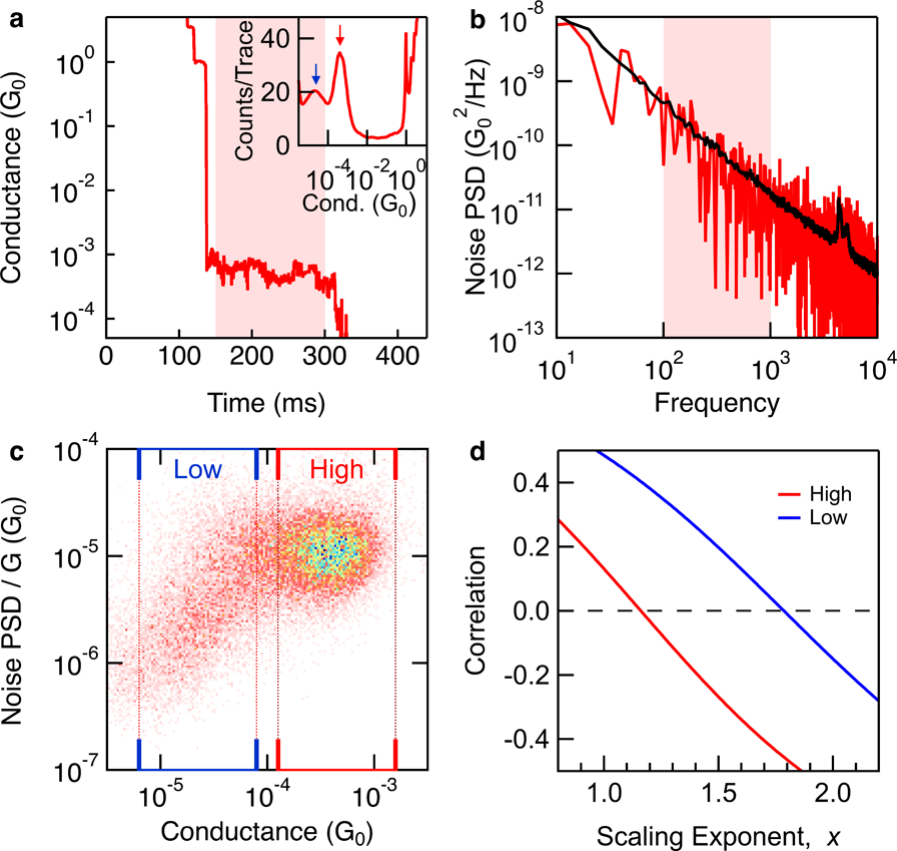
Flicker noise data reproduced in part from Magyarkuti et al.^101^ a) Sample flicker noise measurement trace including a constant-bias hold segment at 100 mV for **pTDA** measured in TCB. Tip–substrate displacement Δ*z* is paused while the junction is held within the highlighted region. Inset: conductance histogram used to identify traces which trap a high (red, single-molecule) or low (blue, π-stacked dimer) conducting junction. b) The red trace shows conductance noise PSD-frequency dependence for the trace shown in panel (a). A clear 1/f signature is observed. The black trace shows the average PSD from 1000 traces where a peak is visible above 1000 Hz due to some instrument noise. c) Normalized noise power (calculated between 100 and 1000 Hz) versus conductance for all traces measured is shown as a two-dimensional plot. The high-conducting junctions have a more circular feature while the low-conducting junctions show a strong conductance dependence in noise power. d) Pearsons’ correlation coefficient against scaling exponent showing that the high-conducting junctions have zero correlation for *x* = 1.16 while the low-conducting junctions have a zero correlation for *x* = 1.78. Reproduced from ref 101. with permission from the Royal Society of Chemistry. Copyright 2018 Royal Society of Chemistry.

Flicker noise power can be calculated for each trace by integrating PSD over the range of 100–1000 Hz; this range is selected specifically for our instrument. Above 1 kHz, we have noise from the electronics that dominate (as shown in the black trace in Figure 12b) and can lead to a roll off of the PSD, and below 100 Hz, mechanical vibrations and AC electrical noise contribute to the measured noise. Each group should determine the best range for their experiments based on the electronics in use and the ambient noise typically present. For instance, if there is acoustic noise at 700 Hz, the range that is integrated should exclude this region. After thousands of traces are collected, the integrated noise power normalized by the average conductance during the hold (*PSD*/*G*) and average conductance (*G*) are calculated for each trace, then compiled into a 2D histogram to observe the scaling relationship. Figure 12c shows an example 2D histogram for **pTDA** where two features are visible, one that is more circular at a high conductance range typical for single-molecule junctions and a more oval feature at a lower conductance range indicating a strong dependence of *PSD*/*G* on conductance. Through measurements of multiple molecules, it was found that *PSD*/*G* scaled as *G* ^*x*^, with the value of *x* depending on the nature of the molecule–electrode interaction. Through-bond coupling resulted in noise power scaling with *G* ^1^, while through-space coupling resulted in noise power scaling with *G* ^2^. It was also observed that π-stacked dimers where each molecule was bound to the Au electrode only one side also showed a scaling exponent *x* ∼ 2.^101^ The relationship between noise power and conductance can be determined by calculating the Pearson’s correlation coefficient between *PSD*/*G* ^x^ and *G*, then determining *x* such that these two variables are uncorrelated (correlation coefficient is zero).^101^ As an example, Figure 12d shows the Pearson’s correlation coefficient against the exponent *x* for both conductance regions shown in Figure 12c. The high conductance region has a scaling relation *G* ^1.16^, which is close to a linear relationship. The low conductance region has a scaling relation *G* ^1.78^, which is closer to a square relationship. This indicated that the lower conducting feature was not due to a through-bond coupled junction but likely due to a π-stacked dimer formed after the single-molecule junction was broken.

### 5.4 Push–Pull Experiments

In addition to the experiments described above, numerous other customizations to the tip–substrate displacement profile and applied bias modulation can be designed to probe a variety of junction phenomena.^107,108^ One such example of an experiment utilizing a nonstandard tip–substrate displacement profile is a “push-pull” experiment reported in Camarasa-Gomez et al.^109^ Data from this publication has been reproduced in Figure 13. In this experiment, a molecular junction is mechanically compressed and expanded by modulating the piezo. Under such modulation, junctions containing a ferrocene derivative shown in Figure 13a were repeatedly stretched and compressed via rotation of the cyclopentadienyl rings about the central Fe atom, altering the molecular confirmation within the junction. As a result, the junction conductance was seen switching between two values depending on the tip–substrate distance. Conductance values were analyzed in the first hold segment at each displacement distance, and the compressed segment was found to have increased conductance by a factor of 6.7 as compared to the stretched segment.

**Figure 13:**
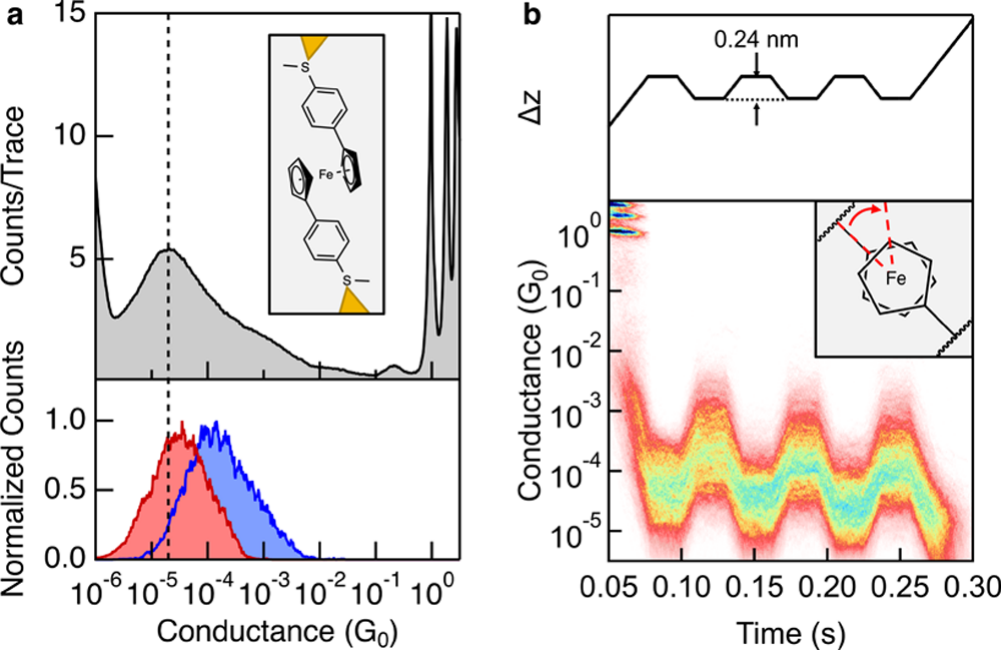
a) Top: 1D histogram of 10000 conductance traces collected for the shown ferrocene derivative at 450 mV bias. Bottom: normalized 1D histograms of conductance values measured at two different tip–substrate displacement distances, with the red curve representing a stretched junction distance and the blue curve representing a compressed junction distance. b) Top: tip–substrate displacement profile used in measurement. Distance is oscillated between two values 0.24 nm apart. Bottom: 2D histogram showing oscillations in conductance according to displacement distance. Inset: molecular rotation observed when junctions oscillate between two configurations. Reproduced from ref 109. Copyright 2020 American Chemical Society.

Push–pull measurements exemplify the versatility of the STM-BJ technique, as they probe how molecular conductance and chemical structure responds to controlled mechanical changes to the junction, rather than looking at response to changes in an applied bias alone. Beyond these experiments, customized displacement profiles, holds, ramps, and bias modulation can be further creatively combined to design measurement protocols tailored to specific chemical and physical questions.

## 6 Conclusion

In this tutorial, we have provided a practical, step-by-step guide to instrument development, experimentation, data interpretation and histogram analysis, as well as possible variations to the standard experiment. We have emphasized hands-on considerations such as tip preparation, noise mitigation, and automation parameters to support researchers and students new to the field, and to promote reproducibility across laboratories. Additional instrumentation can be added to the STM-BJ setup to measure many other properties,^39^ such as bond rupture forces,^110–112^ thermopower,^61–63^ electrochemical phenomena,^22,64,113^ irradiated junctions,^114–117^ and electroluminescence,^71–73^ to name a few. We believe this work will serve as an introduction and a reference point for STM-BJ experiments as the field of single-molecule electronics continues to evolve.

The STM-BJ technique has become an important method for probing charge transport through single molecules. Achieving reproducible, meaningful results requires careful attention to instrument stability, grounding, and potential sources of contamination. A thorough understanding of the features and artifacts observed in conductance-displacement traces is essential, as misidentifying conductance peaks, impurities, and noise signals can lead to misleading conclusions. This tutorial aims not only to improve data quality, but also to encourage critical evaluation of published work in the growing STM-BJ literature.

## References

[1] Joachim C., Gimzewski J. K., Schlittler R. R., Chavy C. (1995). Electronic Transparence
of a Single C60 Molecule. Phys. Rev. Lett..

[2] Reed M. A., Zhou C., Muller C. J., Burgin T. P., Tour J. M. (1997). Conductance
of a molecular junction. Science.

[3] Xu B. Q., Tao N. J. (2003). Measurement
of single-molecule resistance by repeated
formation of molecular junctions. Science.

[4] Choi S. H., Kim B., Frisbie C. D. (2008). Electrical resistance of long conjugated molecular
wires. Science.

[5] Meisner J. S., Kamenetska M., Krikorian M., Steigerwald M. L., Venkataraman L., Nuckolls C. (2011). A Single-Molecule Potentiometer. Nano Lett..

[6] Vezzoli A. (2022). Mechanoresistive
single-molecule junctions. Nanoscale.

[7] Elbing M., Ochs R., Koentopp M., Fischer M., von Hanisch C., Weigend F., Evers F., Weber H. B., Mayor M. (2005). A single-molecule
diode. Proc. Natl. Acad. Sci. U.S.A..

[8] Diez-Perez I., Hihath J., Lee Y., Yu L., Adamska L., Kozhushner M. A., Oleynik I., Tao N. (2009). Rectification and stability
of a single molecular diode with controlled orientation. Nat. Chem..

[9] Lortscher E., Gotsmann B., Lee Y., Yu L. P., Rettner C., Riel H. (2012). Transport Properties of a Single-Molecule Diode. ACS Nano.

[10] Batra A., Darancet P. T., Chen Q., Meisner J., Widawsky J. R., Neaton J. B., Nuckolls C., Venkataraman L. (2013). Tuning Rectification
in Single-Molecular Diodes. Nano Lett..

[11] Capozzi B., Xia J., Adak O., Dell E. J., Liu Z.-F., Taylor J. C., Neaton J. B., Campos L. M., Venkataraman L. (2015). Single-molecule
diodes with high rectification ratios through environmental control. Nat. Nanotechnol..

[12] Dulic D., van der Molen S. J., Kudernac T., Jonkman H. T., de Jong J. J. D., Bowden T. N., van Esch J., Feringa B. L., van Wees B. J. (2003). One-way
optoelectronic switching of photochromic molecules on gold. Phys. Rev. Lett..

[13] Xu B. Q., Li X. L., Xiao X. Y., Sakaguchi H., Tao N. J. (2005). Electromechanical and conductance switching properties
of single oligothiophene molecules. Nano Lett..

[14] Lortscher E., Ciszek J. W., Tour J., Riel H. (2006). Reversible and controllable
switching of a single-molecule junction. Small.

[15] Liljeroth P., Repp J., Meyer G. (2007). Current-induced hydrogen tautomerization
and conductance switching of naphthalocyanine molecules. Science.

[16] Quek S. Y., Kamenetska M., Steigerwald M. L., Choi H. J., Louie S. G., Hybertsen M. S., Neaton J. B., Venkataraman L. (2009). Mechanically
Controlled Binary Conductance Switching of a Single-Molecule Junction. Nat. Nanotechnol..

[17] Zhang K., Wang C., Zhang M., Bai Z., Xie F.-F., Tan Y.-Z., Guo Y., Hu K.-J., Cao L., Zhang S. (2020). A Gd@C82 single-molecule electret. Nat. Nanotechnol..

[18] Li J., Hou S., Yao Y.-R., Zhang C., Wu Q., Wang H.-C., Zhang H., Liu X., Tang C., Wei M. (2022). Room-temperature logic-in-memory
operations in single-metallofullerene
devices. Nat. Mater..

[19] Yao X., Guan L., Maurel F., Lafolet F., Sun X., Lacroix J.-C. (2025). Two-State, Nonvolatile
Memristors and Light-Assisted
Logic-In Operation from Diarylethene-Based Single-Molecule Junctions. J. Am. Chem. Soc..

[20] Liang W. J., Shores M. P., Bockrath M., Long J. R., Park H. (2002). Kondo resonance
in a single-molecule transistor. Nature.

[21] Yu L. H., Natelson D. (2004). The Kondo
effect in C-60 single-molecule transistors. Nano Lett..

[22] Brooke R. J., Jin C., Szumski D. S., Nichols R. J., Mao B.-W., Thygesen K. S., Schwarzacher W. (2015). Single-Molecule Electrochemical Transistor Utilizing
a Nickel-Pyridyl Spinterface. Nano Lett..

[23] Martínez-Blanco J., Nacci C., Erwin S. C., Kanisawa K., Locane E., Thomas M., von Oppen F., Brouwer P. W., Fölsch S. (2015). Gating a single-molecule
transistor with individual atoms. Nat. Phys..

[24] Perrin M.
L., Burzurí E., van der Zant H. S. (2015). Single-molecule transistors. Chem. Soc. Rev..

[25] Chen Z., Grace I. M., Woltering S. L., Chen L., Gee A., Baugh J., Briggs G. A. D., Bogani L., Mol J. A., Lambert C. J. (2024). Quantum interference enhances the performance
of single-molecule transistors. Nat. Nanotechnol..

[26] Li L., Low J. Z., Wilhelm J., Liao G., Gunasekaran S., Prindle C. R., Starr R. L., Golze D., Nuckolls C., Steigerwald M. L. (2022). Highly conducting single-molecule topological
insulators based on mono- and di-radical cations. Nat. Chem..

[27] Deng J.-R., González M. T., Zhu H., Anderson H. L., Leary E. (2024). Ballistic
Conductance through Porphyrin Nanoribbons. J.
Am. Chem. Soc..

[28] Shen S., Shiri M., Mahalingam P., Tang C., Bills T., Bushnell A. J., Balandin T. A., Mejía L., Zhang H., Xu B. (2025). Long-Range
Resonant
Charge Transport through Open-Shell Donor-Acceptor Macromolecules. J. Am. Chem. Soc..

[29] Moreland J., Ekin J. W. (1985). Electron tunneling
experiments using Nb-Sn ‘‘break’’
junctions. J. Appl. Phys..

[30] Zhou C., Muller C. J., Deshpande M. R., Sleight J. W., Reed M. A. (1995). Microfabrication
of a mechanically controllable break junction in silicon. Appl. Phys. Lett..

[31] van
Ruitenbeek J. M., Alvarez A., Pineyro I., Grahmann C., Joyez P., Devoret M. H., Esteve D., Urbina C. (1996). Adjustable
nanofabricated atomic size contacts. Rev. Sci.
Instrum..

[32] Agraıt N., Yeyati A. L., van Ruitenbeek J. M. (2003). Quantum properties of atomic-sized
conductors. Phys. Rep..

[33] Agraït N., Rodrigo J. G., Vieira S. (1993). Conductance
steps and quantization
in atomic-size contacts. Phys. Rev. B.

[34] Evers F., Korytár R., Tewari S., van Ruitenbeek J. M. (2020). Advances
and challenges in single-molecule electron transport. Rev. Mod. Phys..

[35] Gehring P., Thijssen J. M., van der Zant H. S. J. (2019). Single-molecule
quantum-transport
phenomena in break junctions. Nat. Rev. Phys..

[36] Su T. A., Neupane M., Steigerwald M. L., Venkataraman L., Nuckolls C. (2016). Chemical principles
of single-molecule electronics. Nat. Rev. Mater..

[37] Stone I., Starr R. L., Zang Y., Nuckolls C., Steigerwald M. L., Lambert T. H., Roy X., Venkataraman L. (2021). A single-molecule
blueprint for synthesis. Nat. Rev. Chem..

[38] Seideman T. (2003). Current-driven
dynamics in molecular-scale devices. J. Phys.:
Condens. Matter.

[39] Aradhya S. V., Venkataraman L. (2013). Single-molecule junctions beyond
electronic transport. Nat. Nanotechnol..

[40] Muller C. J., van Ruitenbeek J. M., de Jongh L. J. (1992). Experimental observation of the transition
from weak link to tunnel junction. Phys. C:
Supercond..

[41] Krans J. M., Muller C. J., Yanson I. K., Govaert T. C. M., Hesper R., vanRuitenbeek J. M. (1993). One-Atom Point Contacts. Phys.
Rev. B.

[42] Krans J. M., vanRuitenbeek J. M., Fisun V. V., Yanson I. K., Dejongh L. J. (1995). The Signature
of Conductance Quantization in Metallic Point Contacts. Nature.

[43] Scheer E., Agraït N., Cuevas J. C., Yeyati A. L., Ludoph B., Martin-Rodero A., Bollinger G. R., van Ruitenbeek J. M., Urbina C. (1998). The signature of chemical
valence in the electrical
conduction through a single-atom contact. Nature.

[44] Smit R. H.
M., Noat Y., Untiedt C., Lang N. D., van Hemert M. C., van Ruitenbeek J. M. (2002). Measurement of the conductance of a hydrogen molecule. Nature.

[45] Heersche H. B., de Groot Z., Folk J. A., van der Zant H. S. J., Romeike C., Wegewijs M. R., Zobbi L., Barreca D., Tondello E., Cornia A. (2006). Electron transport
through single
Mn-12 molecular magnets. Phys. Rev. Lett..

[46] Kiguchi M., Tal O., Wohlthat S., Pauly F., Krieger M., Djukic D., Cuevas J. C., van Ruitenbeek J. M. (2008). Highly conductive molecular junctions
based on direct binding of benzene to platinum electrodes. Phys. Rev. Lett..

[47] Martin C. A., Ding D., Sorensen J. K., Bjornholm T., van Ruitenbeek J. M., van der Zant H. S. J. (2008). Fullerene-based
anchoring groups
for molecular electronics. J. Am. Chem. Soc..

[48] Tal, O. ; Kiguchi, M. ; Thijssen, W. H. A. ; Djukic, D. ; Untiedt, C. ; Smit, R. H. M. ; van Ruitenbeek, J. M. , Molecular signature of highly conductive metal-molecule-metal junctions. Phys. Rev. B 2009, 80 (8). 10.1103/PhysRevB.80.085427.

[49] Osorio E. A., Moth-Poulsen K., van der Zant H. S. J., Paaske J., Hedegård P., Flensberg K., Bendix J., Bjørnholm T. (2010). Electrical
Manipulation of Spin States in a Single Electrostatically Gated Transition-Metal
Complex. Nano Lett..

[50] Park H., Lim A. K. L., Alivisatos A. P., Park J., McEuen P. L. (1999). Fabrication
of metallic electrodes with nanometer separation by electromigration. Appl. Phys. Lett..

[51] Ralls K. S., Ralph D. C., Buhrman R. A. (1989). Individual-defect
electromigration
in metal nanobridges. Phys. Rev. B.

[52] Bolotin K. I., Kuemmeth F., Pasupathy A. N., Ralph D. C. (2004). Metal-nanoparticle
single-electron transistors fabricated using electromigration. Appl. Phys. Lett..

[53] Baumans X., Lombardo J., Brisbois J., Shaw G., Zharinov V., He G., Yu H., Yuan J., Zhu B., Jin K. (2017). Healing
Effect of Controlled Anti-Electromigration on Conventional
and High-Tc Superconducting Nanowires. Small.

[54] Park J., Pasupathy A. N., Goldsmith J. I., Chang C., Yaish Y., Petta J. R., Rinkoski M., Sethna J. P., Abruna H. D., McEuen P. L. (2002). Coulomb blockade and the Kondo effect in single-atom
transistors. Nature.

[55] Venkataraman L., Klare J. E., Tam I. W., Nuckolls C., Hybertsen M. S., Steigerwald M. L. (2006). Single-Molecule
Circuits with Well-Defined Molecular
Conductance. Nano Lett..

[56] Venkataraman L., Klare J. E., Nuckolls C., Hybertsen M. S., Steigerwald M. L. (2006). Dependence of single-molecule junction
conductance
on molecular conformation. Nature.

[57] Aradhya S. V., Meisner J. S., Krikorian M., Ahn S., Parameswaran R., Steigerwald M. L., Nuckolls C., Venkataraman L. (2012). Dissecting
Contact Mechanics from Quantum Interference in Single-Molecule Junctions
of Stilbene Derivatives. Nano Lett..

[58] Darwish N., Díez-Pérez I., Da Silva P., Tao N., Gooding J. J., Paddon-Row M. N. (2012). Observation
of Electrochemically
Controlled Quantum Interference in a Single Anthraquinone-Based Norbornylogous
Bridge Molecule. Angew. Chem., Int. Ed..

[59] Arroyo C. R., Tarkuc S., Frisenda R., Seldenthuis J. S., Woerde C. H., Eelkema R., Grozema F. C., van der
Zant H. S. (2013). Signatures of quantum interference effects on charge
transport through a single benzene ring. Angew.
Chem., Int. Ed..

[60] Pal A. N., Li D., Sarkar S., Chakrabarti S., Vilan A., Kronik L., Smogunov A., Tal O. (2019). Nonmagnetic single-molecule spin-filter
based on quantum interference. Nat. Commun..

[61] Reddy P., Jang S. Y., Segalman R. A., Majumdar A. (2007). Thermoelectricity in
molecular junctions. Science.

[62] Baheti K., Malen J. A., Doak P., Reddy P., Jang S. Y., Tilley T. D., Majumdar A., Segalman R. A. (2008). Probing the chemistry
of molecular heterojunctions using thermoelectricity. Nano Lett..

[63] Widawsky J. R., Darancet P., Neaton J. B., Venkataraman L. (2012). Simultaneous
Determination of Conductance and Thermopower of Single Molecule Junctions. Nano Lett..

[64] Capozzi B., Chen Q., Darancet P., Kotiuga M., Buzzeo M., Neaton J. B., Nuckolls C., Venkataraman L. (2014). Tunable charge
transport in single-molecule junctions via electrolytic gating. Nano Lett..

[65] Li L., Louie S., Orchanian N. M., Nuckolls C., Venkataraman L. (2024). Long-Range
Gating in Single-Molecule One-Dimensional Topological Insulators. J. Am. Chem. Soc..

[66] Zang Y., Stone I., Inkpen M. S., Ng F., Lambert T. H., Nuckolls C., Steigerwald M. L., Roy X., Venkataraman L. (2019). In Situ Coupling
of Single Molecules Driven by Gold-Catalyzed Electrooxidation. Angew. Chem., Int. Ed..

[67] Zang Y., Zou Q., Fu T., Ng F., Fowler B., Yang J., Li H., Steigerwald M. L., Nuckolls C., Venkataraman L. (2019). Directing
isomerization reactions of cumulenes with electric fields. Nat. Commun..

[68] Orchanian N. M., Guizzo S., Steigerwald M. L., Nuckolls C., Venkataraman L. (2022). Electric-field-induced
coupling of aryl iodides with a nickel(0) complex. Chem. Commun..

[69] Zhang B., Schaack C., Prindle C. R., Vo E. A., Aziz M., Steigerwald M. L., Berkelbach T. C., Nuckolls C., Venkataraman L. (2023). Electric fields
drive bond homolysis. Chem. Sci..

[70] Wang X., Zhang B., Fowler B., Venkataraman L., Rovis T. (2023). Alkane Solvent-Derived Acylation
Reaction Driven by Electric Fields. J. Am. Chem.
Soc..

[71] Paoletta A. L., Hoffmann N. M., Cheng D. W., York E., Xu D., Zhang B., Delor M., Berkelbach T. C., Venkataraman L. (2024). Plasmon-Exciton Strong Coupling in Single-Molecule
Junction Electroluminescence. J. Am. Chem. Soc..

[72] Paoletta A. L., Fung E. D., Venkataraman L. (2022). Gap Size-Dependent Plasmonic Enhancement
in Electroluminescent Tunnel Junctions. ACS
Photonics.

[73] Fung E. D., Venkataraman L. (2020). Too Cool for Blackbody Radiation:
Overbias Photon Emission
in Ambient STM Due to Multielectron Processes. Nano Lett..

[74] Datta, S. Electronic Transport in Mesoscopic Systems; Cambridge University Press, 1995.

[75] Bagrets A. (2013). Spin-Polarized
Electron Transport Across Metal-Organic Molecules: A Density Functional
Theory Approach. J. Chem. Theory Comput..

[76] Blum V., Gehrke R., Hanke F., Havu P., Havu V., Ren X., Reuter K., Scheffler M. (2009). Ab initio molecular simulations with
numeric atom-centered orbitals. Comput. Phys.
Commun..

[77] Arnold A., Weigend F., Evers F. (2007). Quantum chemistry calculations
for
molecules coupled to reservoirs: Formalism, implementation, and application
to benzenedithiol. J. Chem. Phys..

[78] Perdew J. P., Burke K., Ernzerhof M. (1996). Generalized
Gradient Approximation
Made Simple. Phys. Rev. Lett..

[79] Brandbyge M., Mozos J.-L., Ordejón P., Taylor J., Stokbro K. (2002). Density-functional
method for nonequilibrium electron transport. Phys. Rev. B.

[80] Lambert C. J. (2015). Basic concepts
of quantum interference and electron transport in single-molecule
electronics. Chem. Soc. Rev..

[81] Pascual J. I., Méndez J., Gómez-Herrero J., Baró A. M., Garcia N., Landman U., Luedtke W. D., Bogachek E. N., Cheng H. P. (1995). Electrical and mechanical
properties of metallic nanowires:
Conductance quantization and localization. J.
Vac. Sci. Technol. B.

[82] Mosso, N. ; Prasmusinto, A. ; Gemma, A. ; Drechsler, U. ; Novotny, L. ; Gotsmann, B. , Quantized thermal conductance in metallic heterojunctions. Appl. Phys. Lett. 2019, 114 (12). 10.1063/1.5086483.

[83] Kim L., Czyszczon-Burton T. M., Nguyen K. M., Stukey S., Lazar S., Prana J., Miao Z., Park S., Chen S. F., Inkpen M. S. (2024). Low Vapor Pressure Solvents for Single-Molecule Junction
Measurements. Nano Lett..

[84] Pan X., Lawson B., Rustad A. M., Kamenetska M. (2020). pH-Activated
Single Molecule Conductance and Binding Mechanism of Imidazole on
Gold. Nano Lett..

[85] Pan, X. ; Qian, C. ; Chow, A. ; Wang, L. ; Kamenetska, M. , Atomically precise binding conformations of adenine and its variants on gold using single molecule conductance signatures. J. Chem. Phys. 2022, 157 (23). 10.1063/5.0103642. 36550043

[86] Miao Z., Pan X., Kamenetska M. (2024). Conductance
and assembly of quasi-1D coordination chain
molecular junctions with triazole derivatives. Dalton Trans..

[87] Sun N., Zhang Y.-L., Zhou L.-L., Wan Q., Wang Y.-H., Zheng J.-F., Zhou X.-S. (2025). Recent advances
in electrical properties
of single-molecule junctions with nitrogen-based heterocyclic molecules. J. Mol. Struct..

[88] Li T., Bandari V. K., Schmidt O. G. (2023). Molecular Electronics: Creating and
Bridging Molecular Junctions and Promoting Its Commercialization. Adv. Mater..

[89] Daaoub A., Morris J. M. F., Béland V. A., Demay-Drouhard P., Hussein A., Higgins S. J., Sadeghi H., Nichols R. J., Vezzoli A., Baumgartner T. (2023). Not So Innocent After
All: Interfacial Chemistry Determines Charge-Transport Efficiency
in Single-Molecule Junctions. Angew. Chem.,
Int. Ed..

[90] Ward J. S., Vezzoli A., Wells C., Bailey S., Jarvis S. P., Lambert C. J., Robertson C., Nichols R. J., Higgins S. J. (2024). A Systematic
Study of Methyl Carbodithioate Esters as Effective Gold Contact Groups
for Single-Molecule Electronics. Angew. Chem.,
Int. Ed..

[91] Kamenetska M., Koentopp M., Whalley A., Park Y. S., Steigerwald M., Nuckolls C., Hybertsen M., Venkataraman L. (2009). Formation
and Evolution of Single-Molecule Junctions. Phys. Rev. Lett..

[92] Yanson A. I., Bollinger G. R., van den Brom H. E., Agrait N., van Ruitenbeek J. M. (1998). Formation
and manipulation of a metallic wire of single gold atoms. Nature.

[93] Kamenetska, M. ; Widawsky, J. R. ; Dell’Angela, M. ; Frei, M. ; Venkataraman, L. , Temperature dependent tunneling conductance of single molecule junctions. J. Chem. Phys. 2017, 146 (9). 10.1063/1.4973318.

[94] Huo C., Vezzoli A., Vasiljevic N., Schwarzacher W. (2024). Halide adsorption
influences snapback distance in Scanning Tunnelling Microscope break
junctions. Electrochem. Commun..

[95] Ulrich J., Esrail D., Pontius W., Venkataraman L., Millar D., Doerrer L. H. (2006). Variability of Conductance
in Molecular
Junctions. J. Phys. Chem. B.

[96] Peng H. H., Lin C.-H., Tung P.-W., Lin C.-W., Chiang Y.-C., Wang B.-S., Ning T.-H., Ni I. C., Wu C.-I., Chen C.-h. (2026). Interfacial Hopping
Integral as a Predictive Descriptor
for Electron Transport: Saturated Alkane Junctions. J. Am. Chem. Soc..

[97] Park Y. S., Widawsky J. R., Kamenetska M., Steigerwald M. L., Hybertsen M. S., Nuckolls C., Venkataraman L. (2009). Frustrated
Rotations in Single-Molecule Junctions. J. Am.
Chem. Soc..

[98] Kim T., Vázquez H., Hybertsen M. S., Venkataraman L. (2013). Conductance
of Molecular Junctions Formed with Silver Electrodes. Nano Lett..

[99] Hybertsen M. S., Venkataraman L., Klare J. E., Whalley A. C., Steigerwald M. L., Nuckolls C. (2008). Amine-linked single-molecule circuits: systematic trends
across molecular families. J. Phys.: Condens.
Matter.

[100] Gonzalez M. T., Wu S. M., Huber R., van der
Molen S. J., Schonenberger C., Calame M. (2006). Electrical conductance
of molecular junctions by a robust statistical analysis. Nano Lett..

[101] Magyarkuti A., Adak O., Halbritter A., Venkataraman L. (2018). Electronic and mechanical characteristics of stacked
dimer molecular junctions. Nanoscale.

[102] Brisendine J. M., Refaely-Abramson S., Liu Z.-F., Cui J., Ng F., Neaton J. B., Koder R. L., Venkataraman L. (2018). Probing Charge
Transport through Peptide Bonds. J. Phys. Chem.
Lett..

[103] Pan X., Matthews K., Lawson B., Kamenetska M. (2023). Single-Molecule
Conductance of Intramolecular Hydrogen Bonding in Histamine on Gold. J. Phys. Chem. Lett..

[104] Nagahara L. A., Thundat T., Lindsay S. M. (1989). Preparation
and
characterization of STM tips for electrochemical studies. Rev. Sci. Instrum..

[105] Widawsky J. R., Kamenetska M., Klare J., Nuckolls C., Steigerwald M. L., Hybertsen M. S., Venkataraman L. (2009). Measurement
of voltage-dependent electronic transport across amine-linked single-molecular-wire
junctions. Nanotechnol..

[106] Adak O., Rosenthal E., Meisner J., Andrade E. F., Pasupathy A. N., Nuckolls C., Hybertsen M. S., Venkataraman L. (2015). Flicker Noise
as a Probe of Electronic Interaction
at Metal-Single Molecule Interfaces. Nano Lett..

[107] Yao X., Vonesch M., Guan L., Wytko J., Weiss J., Sun X., Lacroix J.-C. (2024). Reliable I/V characteristics and long lifetime of porphyrin-based
single-molecule junctions. J. Mater. Chem. C.

[108] Lee W., Li L., Camarasa-Gómez M., Hernangómez-Pérez D., Roy X., Evers F., Inkpen M. S., Venkataraman L. (2024). Photooxidation
driven formation of Fe-Au linked ferrocene-based single-molecule junctions. Nat. Commun..

[109] Camarasa-Gómez M., Hernangómez-Pérez D., Inkpen M. S., Lovat G., Fung E. D., Roy X., Venkataraman L., Evers F. (2020). Mechanically Tunable Quantum Interference
in Ferrocene-Based Single-Molecule Junctions. Nano Lett..

[110] Xu B., Xiao X., Tao N. J. (2003). Measurements of
Single-Molecule Electromechanical
Properties. J. Am. Chem. Soc..

[111] Frei M., Aradhya S. V., Koentopp M., Hybertsen M. S., Venkataraman L. (2011). Mechanics and chemistry: single molecule
bond rupture
forces correlate with molecular backbone structure. Nano Lett..

[112] Nef C., Frederix P. L., Brunner J., Schönenberger C., Calame M. (2012). Force-conductance correlation in
individual molecular
junctions. Nanotechnol..

[113] Ward J. S., Vezzoli A. (2022). Key advances in electrochemically-addressable
single-molecule electronics. Curr. Opin. Electrochem..

[114] Zhou J., Wang K., Xu B., Dubi Y. (2018). Photoconductance
from Exciton Binding in Molecular Junctions. J. Am. Chem. Soc..

[115] Darwish N., Aragonès A. C., Darwish T., Ciampi S., Díez-Pérez I. (2014). Multi-Responsive Photo- and Chemo-Electrical
Single-Molecule Switches. Nano Lett..

[116] Fung E. D., Adak O., Lovat G., Scarabelli D., Venkataraman L. (2017). Too Hot for Photon-Assisted Transport:
Hot-Electrons
Dominate Conductance Enhancement in Illuminated Single-Molecule Junctions. Nano Lett..

[117] Battacharyya S., Kibel A., Kodis G., Liddell P. A., Gervaldo M., Gust D., Lindsay S. (2011). Optical Modulation
of Molecular Conductance. Nano Lett..

